# Age-Related Changes following In Vitro Stimulation with *Rhodococcus equi* of Peripheral Blood Leukocytes from Neonatal Foals

**DOI:** 10.1371/journal.pone.0062879

**Published:** 2013-05-17

**Authors:** Priyanka Kachroo, Ivan Ivanov, Ashley G. Seabury, Mei Liu, Bhanu P. Chowdhary, Noah D. Cohen

**Affiliations:** 1 Department of Veterinary Integrative Biosciences, College of Veterinary Medicine & Biomedical Sciences, Texas A&M University, College Station, Texas, United States of America; 2 Department of Veterinary Physiology & Pharmacology, College of Veterinary Medicine & Biomedical Sciences, Texas A&M University, College Station, Texas, United States of America; 3 Department of Large Animal Clinical Sciences, College of Veterinary Medicine, Texas A&M University, College Station, United States of America; Institut National de la Santé et de la Recherche Médicale U 872, France

## Abstract

*Rhodococcus equi* is an intracellular bacterium primarily known as an equine pathogen that infects young foals causing a pyogranulomatuous pneumonia. The molecular mechanisms mediating the immune response of foals to *R. equi* are not fully elucidated. Hence, global genomic high-throughput tools like gene expression microarrays might identify age-related gene expression signatures and molecular pathways that contribute to the immune mechanisms underlying the inherent susceptibility of foals to disease caused by *R. equi*. The objectives of this study were 2-fold: 1) to compare the expression profiles at specific ages of blood leukocytes from foals stimulated with virulent *R. equi* with those of unstimulated leukocytes; and, 2) to characterize the age-related changes in the gene expression profile associated with blood leukocytes in response to stimulation with virulent *R. equi*. Peripheral blood leukocytes were obtained from 6 foals within 24 hours (h) of birth (day 1) and 2, 4, and 8 weeks after birth. The samples were split, such that half were stimulated with live virulent *R. equi,* and the other half served as unstimulated control. RNA was extracted and the generated cDNA was labeled with fluorescent dyes for microarray hybridizations using an equine microarray. Our findings suggest that there is age-related differential expression of genes involved in host immune response and immunity. We found induction of genes critical for host immunity against pathogens (MHC class II) only at the later time-points (compared to birth). While it appears that foals up to 8-weeks of age are able to initiate a protective inflammatory response against the bacteria, relatively decreased expression of various other immune-related genes points toward inherent diminished immune responses closer to birth. These genes and pathways may contribute to disease susceptibility in foals if infected early in life, and might thus be targeted for developing preventative or therapeutic strategies.

## Introduction

*Rhodococcus equi* is a gram-positive, facultative, intracellular pathogen that primarily infects macrophages [1]. Although recognized as a cause of disease in people, particularly those that are immunocompromised by HIV or chemotherapeutics, *R. equi* is most commonly recognized as a cause of severe pneumonia in the equine species [2]–[4]. Among horses, the disease occurs almost exclusively among foals, predominately during the first 3 months of life; mature horses are rarely affected unless they have an underlying immunodeficiency [5], [6]. The finding that *R. equi* pneumonia is essentially restricted to foals is likely related to exposure and infection during early life and naïve or diminished immune responses of neonatal foals. Although the age at which foals develop *R. equi* pneumonia remains unknown, epidemiological and clinical evidence indicate that foals are likely infected very early in life [7], [8]. This evidence is consistent with the insidious development of clinical signs in most affected foals [5], and the time required for development of large, pyogranulomatous lesions caused by *R. equi*. Evidence exists that the systemic immune system of newborn foals is diminished relative to more mature foals and horses, including both innate and adaptive responses to *R. equi* [9]–[11]. Thus, the relatively diminished immune responses of newborn foals may predispose them to infection with *R. equi* during early life.

The specific element(s) of immunity that predispose foals to infection with *R. equi* remain ill-defined. The purposes of the study reported here were: 1) to compare gene expression by foal peripheral blood leukocytes stimulated with virulent *R. equi* with gene expression of unstimulated leukocytes, at specific ages during the first 8 weeks of life (i.e., to compare gene expression of stimulated and unstimulated leukocytes within a specific age category); and 2) to compare genes differentially expressed following stimulation of foal leukocytes with *R. equi* during the first 8 weeks of life relative to those differentially expressed following stimulation at birth (i.e., to identify age-related changes in gene expression in response to *R. equi*). Our objective was to identify genes and their related pathways that might contribute to the age-related susceptibility to *R. equi* infection.

## Materials and Methods

### Sample Collection

The protocol for this study was approved by the Texas A&M University Institutional Animal Care and Use Committee (Animal Use Protocol #2006-229). Six healthy Quarter Horse foals born at the Texas A&M University Horse Center were used in this study. All study foals were deemed to be healthy on the basis of results within reference ranges for complete blood counts on day 1 of life and absence of clinical signs throughout the study period. All foals were tested for evidence of passive transfer of maternal immunoglobulins and were found to have serum immunoglobulin concentrations >800 mg/dL using a commercially available immunoassay (SNAP* Foal IgG Test Kit, IDEXX Laboratories, Portland, ME). Approximately 60 mL of blood were collected in 10-mL tubes containing ethylenediamonetetracetic acid (EDTA) via jugular venipuncture from each foal at day 1 (within first 24 h of birth) and at 2, 4, and 8 weeks of age. Blood was immediately processed in the laboratory following collection. The blood was divided into two 30-mL aliquots; each 30-mL aliquot was equally divided into a 6-well tissue culture plate (i.e., 5 mL per well). For 1 aliquot (plate), live, virulent *R.equi* (strain ATCC 33701) suspended in 1 mL of phosphate-buffered saline (PBS) were added to each well at an approximate multiplicity of infection (MOI) of 10, while the other aliquot (plate) had an equivalent volume of PBS added and served as the unstimulated control. The number of bacteria needed to achieve the MOI was based on blood leukocyte concentration as determined using a cell counter (Cellometer Auto T4, Nexcelcom Bioscience, Lawrence, MA). The tissue culture plates were incubated for 2 h at 37°C in 5% CO_2_ with slow rotation. After incubation, blood for each aliquot (*R. equi*-stimulated or unstimulated) was combined, and the leukocytes were isolated and stabilized using a commercial kit (LeukoLOCK Total RNA Isolation System, Ambion, CA), according to the manufacturer’s recommendations.

### RNA Extraction and Amplification

From the isolated and stabilized leukocytes, total RNA was extracted using the LeukoLOCK Total RNA Isolation System (Ambion, CA) according to the manufacturer’s instructions. A DNase I treatment also was performed as a part of the RNA extraction protocol to remove any contaminating genomic DNA. Extracted RNA was quantified with a spectrophotometer (NanoDrop, Thermo Scientific, Wilmington, DE) and the RNA quality was subsequently assessed using a Bioanalyzer (Agilent 2100, Santa Clara, CA).

Due to low quantity of RNA obtained from some of the samples, RNA from all samples was amplified to ensure uniformity in processing samples. A total of 500 ng of RNA was included in each amplification reaction using a commercial kit (RampUP, Genisphere, Hatfield, PA), according to the manufacturer’s protocol.

### Labeling and Microarray Hybridization

For each sample, cDNA was generated from total RNA using Superscript II Reverse Transcription kit (Invitrogen, Carlsbad, CA) and labeled with Cy3 or Cy5 dye via an indirect labeling method utilizing dendrimer technology [12]. Labeling was carried out with the 3DNA Array 900 MPX Expression Array Detection kit (Genisphere, Hatfield, PA).

**Figure 1:**
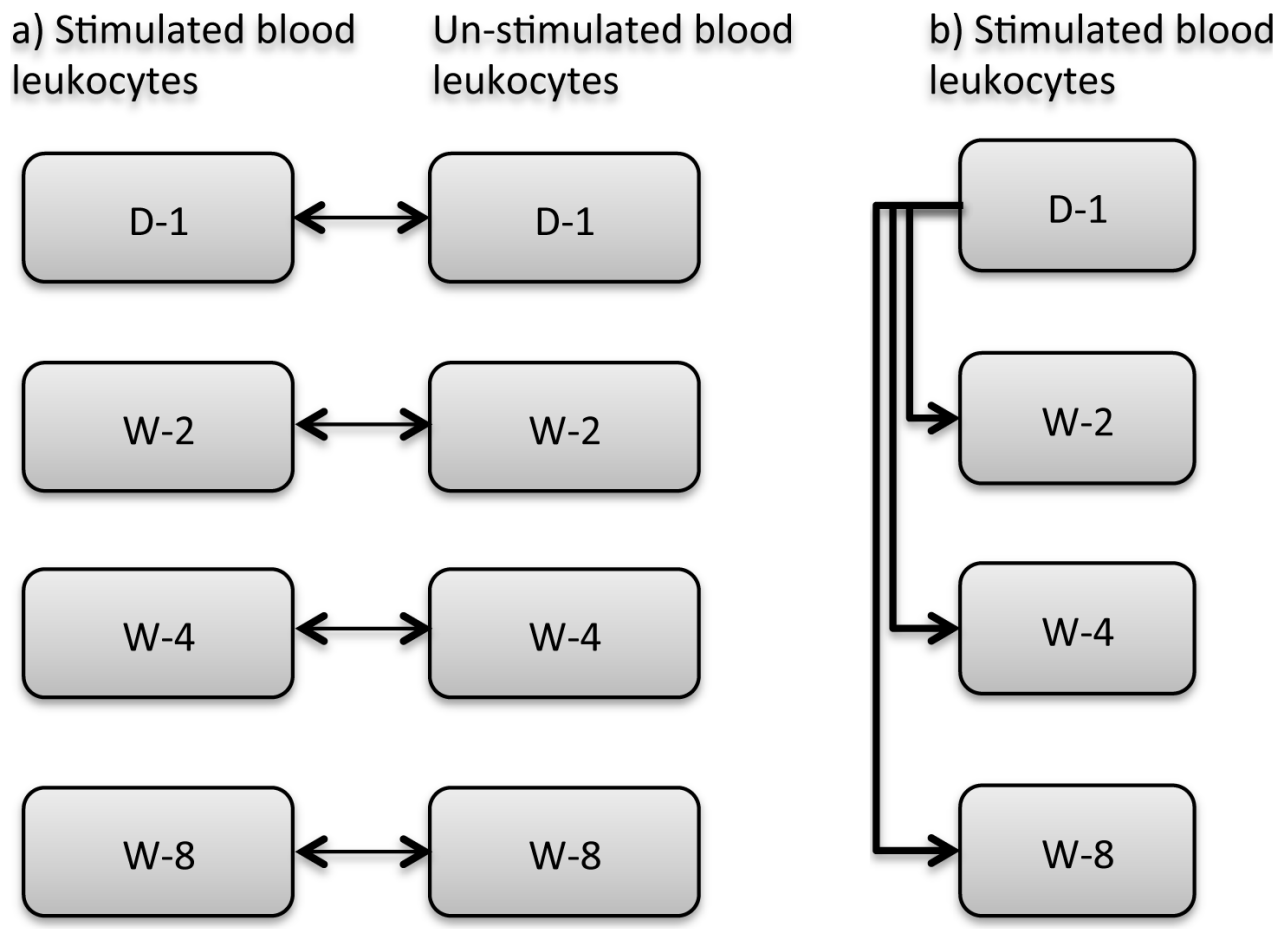
Sample hybridization scheme for microarrays. **a.** Pairwise hybridization of stimulated (with live, virulent *R. equi*) and unstimulated samples was done at each time-point; **b.** Common reference design to hybridize samples from W-2, W-4, and W-8, with D-1 acting as the reference.

Hybridizations were performed with 6 biological replicates at each experimental time-point: day 1 (D-1) and weeks 2 (W-2), 4 (W-4), and 8 (W-8). In order to compare the stimulated blood leukocytes with the unstimulated ones, we performed direct co-hybridization of cDNA from unstimulated samples and the corresponding stimulated samples at each experimental time-point (Figure 1a). Dye-swap was embedded in the biological replicates at each time point such that if the 3 samples of stimulated leukocytes were labeled with Cy3 and hybridized to 3 samples of unstimulated leukocytes labeled with Cy5; the opposite was done with the other 3 stimulated samples and unstimulated samples. Additionally, in order to obtain the temporal expression changes in the *in vitro R. equi* stimulated leukocytes, we utilized the common reference design for microarray hybridizations [13]. The D-1 sample served as the reference for each foal and all other experimental samples from that foal were co-hybridized to it (Figure 1b). Hybridizations were performed in a SureHyb hybridization chamber (Agilent, Santa Clara, CA) at 55°C. Following post-hybridization washes, arrays were scanned with a GenePix 4000B scanner at 5-micron resolution (Molecular Devices, Sunnyvale, CA). GenePix Pro 6.1 software was utilized for raw data acquisition, spot-finding, and quantification of array images.

### Equine 21K Oligonucleotide Microarray

An equine whole genome oligonucleotide array with 21,351 elements developed at Texas A&M University was used for gene expression analysis [14]. This 70-mer oligoarray is one of the most comprehensive arrays available for equine research. The array elements map to various accessions, including those from the UniGene, UniProtKB, Entrez Gene and Non-Redundant Protein Databases. Probes were synthesized (Invitrogen, Carlsbad, CA) and printed onto amino-silane-coated slides (Corning Incorporated, Corning, NY). The equine oligoarray data are compliant with MIAME standards and the array data have been submitted to the NCBI Gene Expression Omnibus (GEO) database (accession number GSE45004).

### Expression Analysis

Differential expression analysis of microarray data was conducted using Bioconductor’s LIMMA package running in the R statistical software environment [15]. Background correction of raw intensities was performed using the normexp correction method with offset 50 [16]. Subsequently, the background corrected intensities were normalized using printip-loess normalization (within array normalization) and aquantile normalization was used for across array normalization was performed [17]. To account for the multiple comparisons, the false discovery rate correction method of Benjamini and Hochberg was used [18]. Linear models [15] were used to identify differentially expressed (DE) genes in the stimulated leukocytes (temporal expression) for the 3 comparisons of interest: a) day 1 versus week 2 (C1); b) day 1 versus week 4 (C2); and, c) day 1 versus week 8 (C3). Similarly, linear models were fit to obtain DE genes between the stimulated and the unstimulated leukocytes at each time-point (D-1, W-2, W-4, W-8). Genes were considered significantly differentially expressed if they had an adjusted p-value <0.05 and fold-change >1.5.

### Functional Analysis

Functional gene ontology analysis was performed using the web-based tool known as DAVID (The **D**atabase for **A**nnotation, **V**isualization and **I**ntegrated **D**iscovery version 6.7; accessible at http://david.abcc.ncifcrf.gov/) [19], [20]. Gene ontology analysis was performed using the list of differentially expressed genes to obtain statistically significant biological processes. In order to calculate the statistical significance, DE genes for each comparison were compared to all the genes present on our equine microarray. Statistical significance cutoff of 0.05 was considered and Benjamini-Hochberg method was used for multiple test adjustment.

### Quantitative Real-Time PCR

To demonstrate the validity of gene expression results obtained by microarray, quantitative real-time PCR (RT-PCR) was performed on 4 genes selected for their biological importance and that were significantly differentially expressed (fold-change >1.5 and p-value<0.05) in our microarray results. Each gene was tested in duplicate along with a housekeeping gene control, beta actin (*ACTB*). Total RNA was directly reverse transcribed to cDNA using the SuperScript® VILO™ cDNA Synthesis Kit (Invitrogen, Carlsbad, CA) and subsequently amplified using gene-specific primers and master mix by RT-PCR in a single-step reaction. Serial dilutions of RNA from the reference sample were used to generate relative standard curves and test the amplification efficiency of each primer set. For each qPCR assay, ∼100 ng of total RNA were used in a 25-µl reaction with 1x Universal SYBR® Green Master Mix (Applied Biosystems, Carlsbad, CA) and 300 nM primers and amplified on a LightCycler 480 (Roche Diagnostics, Indianapolis, IN). The primer sets used to amplify the genes were designed using Primer3 software (http://frodo.wi.mit.edu/primer3/) and are listed in **Table S1 in File S1**.

To validate the results obtained via microarray analysis, the expression levels of 4 selected genes were determined using RT-PCR: tumor necrosis factor (TNF), receptor-associated factor 3 *(TRAF3*), interleukin 1 beta *(IL1B),* interferon gamma *(IFN-γ)*, and nuclear factor of kappa light polypeptide gene enhancer in B-cells inhibitor alpha *(NFKBIA)*. The fold-changes in treated sample with respect to control were calculated using Pfaffl also known as delta-delta Ct method [21]. A Mann-Whitney U test was used to test the statistical significance of mRNA induction values and the test was performed using the online program available at http://elegans.som.vcu.edu/~leon/stats/utest.html.

## Results

### Number of Differentially Expressed Genes

Comparison of the stimulated versus unstimulated peripheral blood leukocytes at D-1 showed that 125 genes were differentially expressed, of which 89 were up-regulated and 36 down-regulated (**Table 1** **, D-1**). A similar comparison for W-2 led to the identification of 127 DE genes, with 92 up-regulated and 35 down-regulated genes (Table 1, W-2). At W-4, 73 genes were differentially expressed with 35 up-regulated and 38 down-regulated genes (**Table 1** **, W-4**). Finally, at W-8 there were 135 genes that were differentially expressed, of which 92 were up-regulated and 43 down-regulated (**Table 1** **, W-8**). A list of all DE genes is presented in tables S5–S8. The listed DE genes were common to all foals.

**Table 1:** Differentially expressed (DE) genes.

Comparison type	Time-point	DE genes	Total DE genes
Stimulated versus unstimulated leukocytes	Day1 (D1)	Up: 89 Down: 36	125
	Week-2 (W2)	Up: 92 Down: 35	127
	Week-4 (W4)	Up: 35 Down: 38	73
	Week-8 (W8)	Up: 92 Down: 43	135
Temporal changes in stimulated leukocytes	Day1 vs. week-2 (C1)	Up: 30 Down: 72	102
	Day1 vs. week-4 (C2)	Up: 83 Down: 109	192
	Day1 vs. week8 (C3)	Up: 75 Down: 73	148

List of differentially expressed genes for each comparison is shown in the table. Genes were considered differentially expressed with P value<0.05 and fold-change >1.5. C1: comparison 1, C2: comparison 2, C3: comparison 3.

Likewise, when D-1 was compared to each of the remaining 3 time-points (W-2, W-4, and W-8) for the stimulated peripheral blood leukocytes, differentially expressed genes were identified for each indicating temporal changes in the gene expression profile. There were 102 DE genes for comparison C1 (D-1 vs. W-2), with the highest number of DE genes for comparison C2 (D1 vs. W-4; 192 genes), and finally C3 (D1 vs. W-8) with 148 DE genes. A list of all DE genes following *R. equi* stimulation is presented in the Tables S9–S11. The listed DE genes were common to all foals.

**Figure 2:**
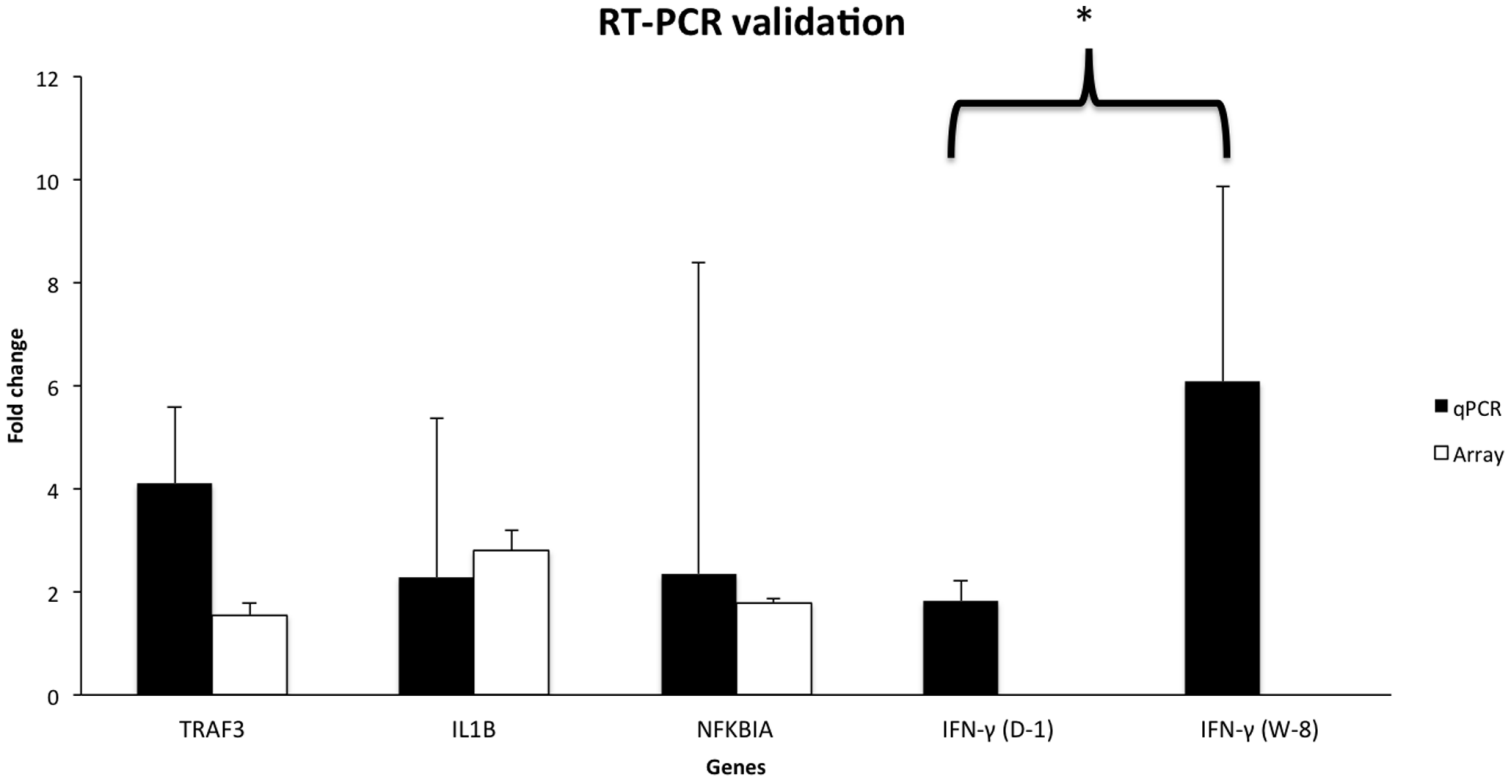
Real-time PCR validation of microarray results. The y-axis represents the fold-change of the selected genes for validation using real-time PCR (white bars) and microarray (black bars). The Y-axis represents fold-change of selected genes by real-time PCR (delta-delta Ct method) and by microarray (normalized log2 (Cy5/Cy3)). * represents significant (P<0.05) difference in fold-change of IFN-γ at W-8 compared to D-1. IFN-γ has no corresponding black bar representing microarray data, as this gene was not printed on the microarray. The error bars indicate standard error of the mean (SEM) which was calculated as SEM = SD/√n, where SD represents standard deviation and n is the sample size.

To validate the results obtained via microarray analysis, the expression levels of 4 selected genes were validated using RT-PCR: *TRAF3*, *IL1B, IFN-γ*, an*d NFKBIA*. The up-regulation of all 4 genes was confirmed at D-1 (**Figure 2**). Compared to unstimulated cells, increased expression of *IFN-γ* was found in stimulated cells at D-1 and W-8. In the stimulated leukocytes, *IFN-γ* mRNA expression was induced more at W-8 than D-1, and this increase was found to be statistically significant with P<0.05 (**Figure 2**).

### Functional Analysis

#### Stimulated versus unstimulated blood leukocytes

We performed functional analysis on the differentially expressed genes associated with each time-point. It appeared from our results that bacterial stimulation of leukocytes induced expression of various immune-related genes. The *in vitro R. equi* stimulation led to modulation of genes involved in cytokine and chemokine signaling, host innate immunity and defense, apoptosis, macrophage-mediated immunity, and various other processes listed in **Table 2**.

**Table 2:** Functional analyses of differentially expressed genes.

	Day-1	Week-2	Week-4	Week-8
**Cytokine and chemokine mediated signaling** **pathway**	CCL20, IFNA5, IL1RN, IL1B,IL1A, TRAF3	CXCL3, IL1RN, IL1B, IL1A, CXCL10	CXCL2, IL1RN, IL1B,IL1A	CCL20, IFNA5, CXCL2, IL1RN, IL1B, IL1A, CXCL10
**Ligand-mediated signaling**	CCL20, INS-IGF2, IFNA5, EDN2, IL1RN, IL1B, IL1A	CXCL3, EDN2, IL1RN, IL1B, IL1A, CXCL10	EDN2, CXCL2, LILRB4, IL1RN, IL1B, IL1A	CCL20, INS-IGF2, IFNA5, EDN2, CXCL2, IL1RN, IL1B, IL1A, CXCL10
**Apoptosis**	DDX58, HIP1R, IFNA5, NFKBIA, IL1B, BIRC3, TRAF3	DDX58, CUL3, NFKBIA, IL1B, BIRC3		DDX58, CUL3, IFNA5, NFKBIA, IL1B, BIRC3
**Immunity and defense**	KLRB1, CCL20, IFNA5, IL1RN, NFKBIA, IL1B, TFCP2, HAGHL, UBP1, PTAFR, IL1A	CXCL3, IL1RN, NFKBIA, IL1B,TFCP2, UBP1, PTAFR, IL1A,SOD2, CXCL10	CXCL2, IL1RN, IL1B, TFCP2, PLAU, IL1A	KLRB1, CXCL2, IL1RN, NFKBIA, TFCP2, UBP1, SOD2, CXCL10, CCL20, IFNA5, IL1B, IL1A, PTAFR, PLAU, GBP1
**NF-kappaB cascade**	IL1RN, IL1B, IL1A, IFNA5,NFKBIA, TRAF3, BIRC3, TNFAIP3	IL1RN, IL1B, IL1A, BIRC3	IL1RN, IL1B, IL1A,BIRC3	IL1RN, IL1B, IL1A, IFNA5, CXCL2, NFKBIA, TRAF6, BIRC3
**Macrophage-mediated immunity**	IFNA5, IL1B, IL1A	CXCL3, IL1B, IL1A, CXCL10	CXCL2, IL1B, IL1A	IFNA5, CXCL2, IL1B, IL1A, GBP1, CXCL10
**Cell surface receptor mediated signal transduction**	GNAI3, INS-IGF2, EDN2, IL1RN, GPR109A, RASGEF1B, OXTR, RALGDS, CCL20, IFNA5, IL1B, GNB3, IL1A, PTAFR, TRAF3	CXCL3, EDN2, OR13D1, IL1RN,RASGEF1B, IL1B, TPR, GRB7,GPR31, PTAFR, IL1A, CXCL10	OR1A1, EDN2, CXCL2, IL1RN, IL1B, IL1A	GPR84, INS-IGF2, EDN2, GPR109A, CXCL2, NPBWR2, IL1RN, RASGEF1B, CXCL10, CCL20, IFNA5, IL1B, IL1A, PTAFR, RHOG, GPR31
**G-protein mediated signaling**	GNAI3, EDN2, GPR109A, OXTR, GNB3, RALGDS, PTAFR			GPR84, EDN2, GPR109A, NPBWR2, GPR31, RHOG, PTAFR
**Signal transduction**	GNAI3, INS-IGF2, EDN2, IL1RN, GPR109A, RASGEF1B, NFKBIA, OXTR, RALGDS, CCL20, IFNA5, IL1B, GNB3, CLINT1, IL1A,PTAFR, TRAF3			GPR84, INS-IGF2, EDN2, GPR109A, IL1RN, CXCL2, NPBWR2, RASGEF1B, NFKBIA, CXCL10, CCL20, IFNA5, RSPH3, IL1B, PLAU, IL1A, PTAFR, RHOG, GPR31
**Cell proliferation and differentiation**		CABP2, CXCL3, STAMBPL1,NFKBIA, IL1B, IL1A, CXCL10		
**Cell structure and motility**				VAPB, CXCL2, NPBWR2, KIF21A, RHOG, PLAU, PTAFR, CXCL10
**Intracellular protein traffic**				LMBR1L, OLR1, VAPB, RSPH3, NFKBIA, KIF21A, SNX10
**Interferon-mediated Immunity**				IFNA5, GBP1, CXCL10

Biological processes associated with differentially expressed genes when *R. equi*-stimulated leukocyte expression was compared to unstimulated leukocytes at day 1 and week 2, 4, and 8.

The genes up-regulated in the stimulated leukocytes (compared within age to unstimulated) appeared to be various up-and down-stream components of Toll-like receptor (TLR) signaling (**Figure S1in File S1**) and the NF-κB activation cascade (**Figure S2 in File S1**). Soon after birth (D-1), we observed modulation of genes involved in nuclear factor-kappaB (NF-κB) signaling in response to ligation of TLRs or interleukin-1 receptor (IL-1R) (**Figure S1 and S2 in File S1**). Several genes at each time-point were present at the various stages of the NF-κB pathway: a) genes that belong to ligands and receptors, viz., *IL1A, IL1B, IL1RN* (D-1, W-2, W-4, W-8); and b) downstream signaling, viz., *TNFAIP3 (A20), TRAF3* (D-1), *TRAF6* (W-8), *NFKIBIA* (D-1, W-2, W-8), and *BIRC3* (D-1, W-2, W-4, W-8).

A variety of chemokines was induced across the time-points in stimulated cells compared to unstimulated: chemokine (C-C motif) ligand 20 (*CCL20)* at D-1; chemokine (C-X-C) motif ligand 3 (*CXCL3)* and *CXCL10* at W-2; *CXCL2* at W-4; and *CCL20*, *CXCL2*, and *CXCL10* at W-8. The biological processes associated with down-regulated gene expression by *R. equi* stimulation on D-1 were involved in wound healing and blood coagulation. On D-1, we observed down-regulation of genes involved in coagulation and platelet activation viz., signal peptide, CUB domain, EGF-like 1 (*SCUBE1)*, selectin P (*SELP)*, and phospholipid scramblase 1 (*PLSCR1*); however, at older ages (W-2 and W-4) there was evidence of up-regulation of genes associated with coagulation and platelet activation viz., plekstrin (*PLEK)* and platelet-activating factor receptor (*PTAFR)* at W-2, and plasminogen activator, urokinase *PLEK* and plasminogen activator, urokinase (*PLAU)* at W-4. Genes like L-plastin (*LCP1*) and L-selectin (*SELP*) that are involved in host immunity against pathogens were also found to be down-regulated at D-1.

The results suggest that stimulation with *R. equi* leads to activation of elements involved in pro-inflammatory pathways. However, it also appears that young foals at D-1 have a reduced repertoire of chemokines and lowered expression of some genes involved in responding to pathogens (e.g., *SELP*).

**Figure 3:**
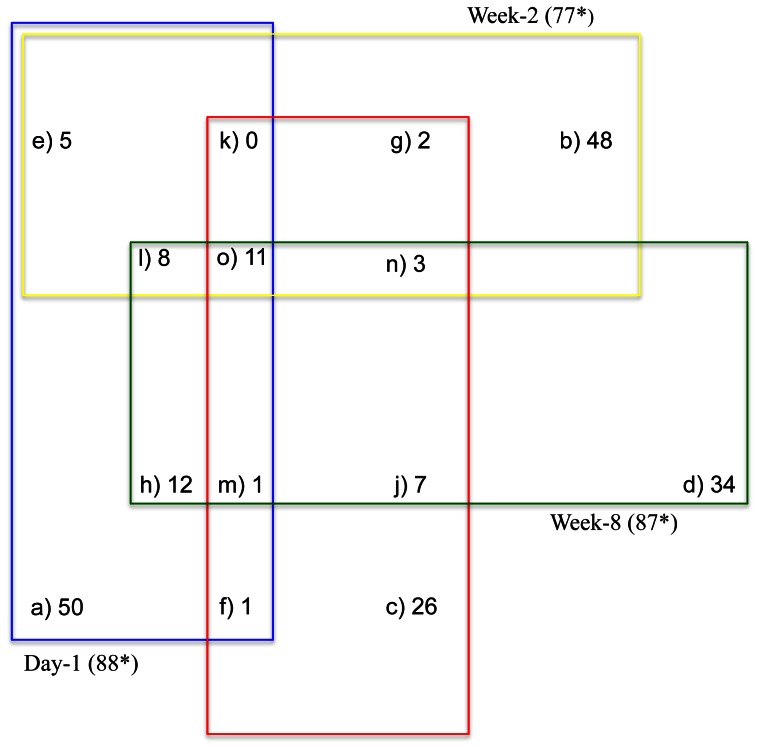
Venn diagram for pairwise comparison of the gene expression profile of stimulated versus unstimulated leukocytes. The 4 time-points are designated in the diagram as 1: day 1, 2: week 2, 3: week 4, 4: week 8. The letters represent: a-genes found in 1 only; b-genes found in 2 only; c-genes found in 3 only; d-genes found in 4 only; e-genes in 1&2 only; f-genes in 1&3 only; g-genes in 2&3 only; h-genes in 1&4 only; i-genes in 2&4 only; j-genes in 3&4 only; k-genes in 1,2,3 only; l-genes in 1,2,4 only; m-genes in 1,3,4 only; n-genes in 2,3,4 only; and, o-genes in 1,2,3, and4. *Only genes with an annotated accession were used for generating this figure.

In order to identify the genes that were unique for each experimental time-point, we compared the list of DE genes across all 4 time-points and obtained the intersection and union of the gene sets (**Figure 3**). The expression profile at each time-point appeared to be largely distinct with very few common genes as depicted in **Figure 3**. Out of the 88 (D-1), 88 (W-2), 51 (W-4), and 87 (W-8) genes differentially expressed at each time-point, only 11 genes were identified at all 4 times indicating that the expression profile was largely unique for each of the 4 time-points. This small fraction of genes commonly induced by *R. equi* stimulation among the various ages (time-points) was consistent with our findings of age-related differences in the expression profile of neonatal immune cells described in the ensuing section.

#### Temporal changes in stimulated blood leukocytes

We also performed functional analysis of the genes differentially expressed in response to stimulation at ages 2, 4, and 8 weeks relative to stimulated responses on D-1 (i.e., comparisons C1, C2, and C3). For comparison C1, the biological processes (GO terms) associated with the up-regulated genes were primarily those involved in antigen processing and presentation and host defense response, including MHC class II presentation associated with CD4+ T cell responses (**Table S2a in File S1**). For the C2 comparison, the up-regulated genes were associated with protein and ion transport (**Table S3a in File S1**). For the C3 comparison, the up-regulated genes were primarily associated with antigen processing (via MHC class II) and presentation and protein metabolism (**Table S4a in File S1**). Down-regulated genes for each of the comparisons (C1, C2, and C3) appeared primarily to involve lipid metabolism and transport.

**Figure 4:**
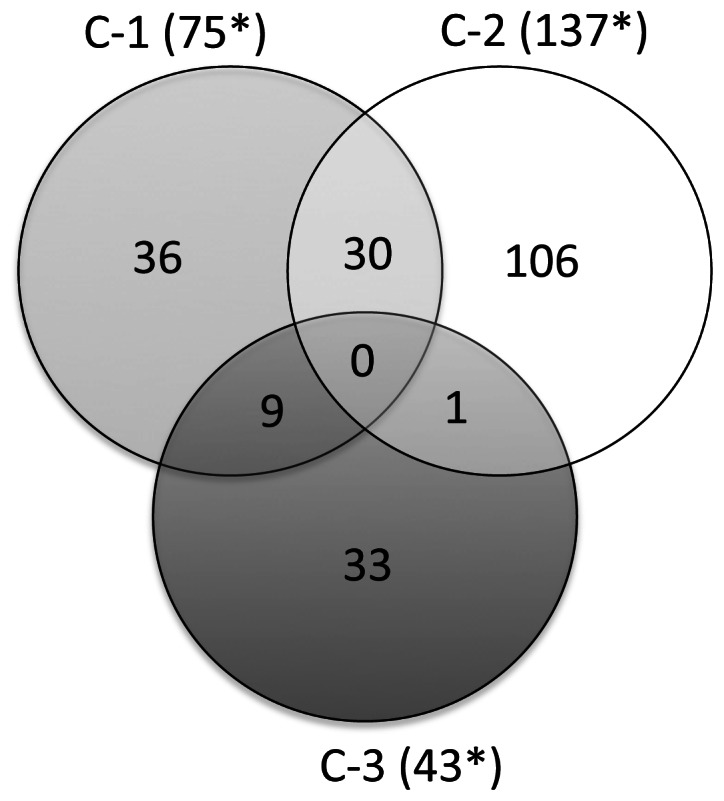
Venn diagram for temporal gene expression changes. This Venn diagram depcts DE genes unique to each comparison: day1-week2 (C1); day1-week4 (C2); and, day1-week8 (C3). *Only genes with an annotated accession were used for generating this figure.

We compared the list of DE genes obtained for each temporal comparison and found the groups of DE genes that were common for 2 or more of the comparisons. (**Figure 4**). The expression profiles of the 3 comparisons (C1, C2, and C3) were largely distinct, with the largest overlap between C1 and C2 (30 genes). None of the genes were present in all 3 comparisons, highlighting the uniqueness of the expression profile at each age.

## Discussion

The gene expression profile of the stimulated leukocytes compared to the unstimulated ones appeared to be largely unique for each time-point. Across time-points, stimulation with *R. equi* induced genes in leukocytes that were involved in defense mechanisms such as inflammation. More importantly, we observed age-related differences in expression of genes involved in host immune responses highlighting diminished immunity during early life of foals. We found induction of genes critical for host immunity against intracellular pathogens such as *R. equi* (viz., MHC class II) only at the later time-points (relative to the day of birth) [22], [23]. Moreover, compared to the unstimulated cells, at birth the stimulated leukocytes displayed reduced expression of genes involved in the various components of innate and adaptive immunity (e.g., down-regulation of genes associated with host immune response such as IFN-γ production at D-1). Therefore, results of this study suggest age-dependent differences in immunity as a plausible underlying factor contributing to susceptibility of foals to *R. equi*.

### Stimulated Responses of Blood Leukocytes at Each Age-Point

When the expression profiles of the stimulated leukocytes were compared to the unstimulated ones within age, a pattern emerged across all 4 time-points. The central theme that surfaced was that bacterial stimulation led to induction of molecular mechanisms involved in the activation of innate immune responses and inflammation via activation of components of the NF-κB cascade (**Figure S2,** **Table 2**).

NF-κB is a critical regulator of host innate immune response and activates inflammatory and cell-survival pathways [24]. Our results suggest that *in vitro R. equi* stimulation possibly leads to activation of NF-κB via TLR signaling or induction by IL-1α (the gene product of *IL1A*)/IL-1β (the gene product of *IL1B*) ligand interaction with IL-1 receptor to initiate inflammatory pathways. Activation of NF-κB leads to secretion of pro-inflammatory cytokines like interleukin IL-1α and IL-1β. The products of *IL1A* and *IL1B* are pyrogens and capable of inducing fever [25]. Fever is a non-specific host response to elevate the body temperature that may help protect against infections with bacteria, including *R. equi*. The products of the *IL1A* and *IL1B* genes are also involved in innate responses including up-regulation of endothelial adhesins for neutrophils, induction of acute-phase proteins, and early recruitment of inflammatory cells to the sites of infection [26]. These innate responses may further help protect foals against infection against *R. equi* in a non-specific manner. Innate immune responses play a direct protective role against *R. equi* infections in mice [27] and also likely serve as an important bridge to protective adaptive immune responses [10].

Modulation of genes involved in TLR signaling that leads to activation of NF-κB was observed (**Figure 2**). Activation of TLR2 and TLR9 and downstream signaling are considered to be involved in response to *R. equi* infection [10], [11]. TLR9 senses unmethylated CpG DNA of viruses and bacteria, and induces interferon IFN-α expression from plasmacytoid dendritic cells (pDC’s) [28]. Consistent with TLR9 activation, we suggest that *R. equi* stimulation activated TLR9 signaling via up-regulation of *TRAF3* expression leading to an increased expression of *IFNA5*; however, we lack translational (protein-level) evidence to corroborate our gene expression data.

Chemokines are a subset of cytokines capable of chemoattraction of immune cells. A variety of chemokine genes were induced across the time-points: *CCL20* on D-1; *CXCL3* and *CXCL10* at W-2; *CXCL2* at W-4; and, *CCL20*, *CXCL2*, and *CXCL10* at W-8. CCL20 is important during the initiation of an immune response [24] and is involved in chemotaxis of immature dendritic cells and effector T- and B-lymphocytes [29]. *CXCL2* is important for neutrophil recruitment, and neutrophils are important for protection against infection with *R. equi* [27]. Induction of *CXCL10* is involved in activation and recruitment of CD4+ and CD8+ T-cells [30]. CD4+ T-cells are critical in effective clearance of *R. equi* [31], making induction of *CXCL10* a key factor in host immune response.

CXCL2 appears to be involved as an activating component of innate immunity while CXCL10 is predominantly involved in inducing adaptive immunity via T-lymphocyte recruitment. The variation in chemokine profiles among time-points reveals that fewer chemokine genes are induced in foals close to birth, and as they age (by week 8), a wider spectrum of chemokines having critical roles in immune responses to infection are induced in response to stimulation with *R. equi*.

The chemokine CXCL10 is involved in activation of effector T-cells critical for *R. equi* clearance and was induced at later time-points but not at D-1. The expression of this chemokine is induced by IFN-γ [32]. Evidence exists that foals are deficient in producing IFN-γ at birth and that its levels increase as foals age [9], [33]. Thus, it is possible that the induction of *CXCL10* and another interferon-inducible gene such as guanylate binding protein 2 *(GBP2)* at W-8 was driven by increased INF-γ expression. Although the gene for INF-γ was not included on our microarray, we were able to demonstrate using RT-PCR that there was significantly increased expression of *IFN-γ* at W-8 compared to D-1; this finding supports the possibility of *CXCL10* and *GBP2* expression having been influenced by *IFN-γ* expression, and substantiates previous reports regarding age-related differences of increased expression of *IFN-γ* as neonatal foals age [9], [33]. Interestingly, *GBP2* belongs to the GTPase gene family which has a putative role in resistance to intracellular pathogens [34].

Down-regulation of various other genes possibly points toward diminished immunity of foals at birth. Alternatively, repression of these immune-related genes may suggest microbial attenuation of host immune response at younger ages. For example, down-regulation of L-plastin (*LCP1*) and L-selectin (*SELP*) was observed at D-1. L-plastin has a role in T-cell activation due to its role in the actin cytoskeleton re-arrangement that is critical for TCR signaling. Loss of function of L-plastin in neutrophils leads to defects in activation of respiratory burst, and down-regulation of this gene impairs T-cell responses to antigen manifested by reduced production of IFN-γ and IL-17 [35]. Our results indicate reduced expression of the *SELP* gene in stimulated leukocytes on D-1. Inhibition of *SELP* expression during *R. equi* infection has been suggested to decrease the maturation and responsiveness of dendritic cells, thereby dampening the host response to this pathogen [36]. Thus, *SELP* down-regulation at D-1 could suggest either a deficiency of the host response to *R. equi* or modulation of host immunity by the pathogen. Similarly, at D-1 we observed down-regulation of various genes involved in coagulation and platelet activation (viz., *SCUBE1, SELP, and PLSCR1*), whereas at older ages (W-2 and W-4) there was evidence of up-regulation of genes associated with coagulation and platelet activation (viz., *PLEK and PTAFR* at W-2 and *PLEK and PLAU* at W-4). Platelet activation may have relevance to *R. equi* infections in foals. Thrombocytosis occurs in association with infections, including foal pneumonia [37]. Moreover, in mycobacterial infection of lungs, platelets may have a protective role wherein the aggregation of platelets results in obstruction of blood vessels around the foci of infection that prevents the spread of bacteria [38].

We identified that *R. equi* exposure of immune cells leads to activation of various components of TLR and NF-κB cascade. Inability of a foal to activate such inflammatory responses early in life may increase their susceptibility to *R. equi* infection. We also identified genes that appear to be associated with age-related differences in immune response. Genes down-regulated at birth such as *IFN-γ*, *CXCL10* and other chemokines, *SELP* and *LCP1* could result in increased susceptibility to *R. equi* infection. Novel drugs and vaccines administered early in life can thus be designed and evaluated for their ability to enhance protective immune responses associated with these genes in young foals.

### Temporal Changes in Gene Expression

Very few genes induced by *R. equi* stimulation were common among ages (**Figure 4**), suggesting that the expression profile at each time-point was distinct. Nevertheless, compared to a 1-day-old foal with naïve immunity, changes in the expression profile over the ensuing 8 weeks demonstrated a theme of immune development, as manifested by up-regulation of a variety of immune-related genes at later time-points. Collectively, these findings suggested that immune function was diminished on D-1 relative to older ages.

Compared to the changes in expression profile of stimulated leukocytes at D-1, various genes involved in immune response and inflammation were up-regulated at later time-points, such as MHC class-II genes (*HLA-DRA*, *HLA-DQB1*) and indoleamine 2,3 dioxygenase (*INDO*). Up-regulation of MHC class II genes at W-2 and older is consistent with the observed increase in MHC class II lymphocytes with age during the first month of life of foals [39]. At birth, reduced MHC II class antigen presentation can reduce activation of class II-restricted CD4+ T-cells [40], and evidence exists that CD4+ cells are critical in clearance of *R. equi* infection in mice and horses [31], [36]. Thus, a reduced expression of MHC class II genes in foals at birth could contribute to increased disease susceptibility. Relative to D-1, *INDO* expression was also up-regulated at W-2, W-4, and W-8 in stimulated leukocytes. The *INDO* gene has been reported to have a role in defense against intracellular pathogens due to the ability of the encoded enzyme to restrict pathogen growth either by depletion of tryptophan or by dampening inflammatory responses [41].

There are various studies that support a critical role of IFN-γ in the clearance of *R. equi*. Unfortunately, the IFN-γ gene was not represented on our microarray and therefore we have no direct evidence supporting the age-dependent induction [9], [33]. However, as indirect evidence we did observe down-regulation of *CCL20* across later time-points relative to D-1. It has recently been proposed that chemokine CCL20 has a role early in infection after which its levels are down-regulated by induction of IFN-γ [42]. Furthermore, MHC class-II genes which are interferon-inducible, were up-regulated only at later time-points compared to their expression at birth. Our RT-PCR data support this observation, since we found mRNA levels of *IFN-γ* to be significantly higher at W-8 than D-1.

Age-related modulation towards lowered expression of genes involved in lipid transport and metabolism was observed for all 3 stimulated response comparisons (C1, C2, and C3). Lipid metabolism is a major source of energy for mycobacteria and, if a similar mechanism exists in *R. equi*, then down-regulation of lipid transport and metabolism genes is perhaps modulated to reduce survival of the pathogen.

This study is one of the first to use a whole genome expression array to understand foal immune responses to *R. equi*. However, we would also like to address some of the limitations of this study. One of the shortcomings of this study was that the mRNA for the expression analysis was obtained after only a single time-period (viz., 2 h) of *in vitro* incubation of peripheral blood leukocytes with *R. equi*. Previous studies from our laboratory have indicated that significant mRNA expression of key immune-related cytokines (e.g., IFN-γ, IL-6, and IL12p40) was obtained by stimulating foal peripheral blood mononuclear cells and neutrophils with *R. equi* for 2 h [11], [43]. Unpublished data from this study also suggested that mRNA expression of various cytokines using this short incubation period was not significantly lower than longer incubations (4, 8, 12, or 24 h). While our unpublished results revealed that longer stimulation times generally yielded higher expression of some cytokines, it also affected the quality and quantity of RNA extracted. Therefore, we elected to use a 2 h incubation period for this study.

Another limitation of this study is that the experimental design makes it difficult to dissect the effects of maturation in response to natural exposure from deficiency. Although *in vitro* stimulation of blood leukocytes from healthy foals was performed, the foals would have been exposed to environmental *R. equi* isolates; thus, the age-related differences in expression profile we observed could simply reflect the transition of young foals from a naïve, unexposed state to an exposed state. *Rhodococcus equi* is widespread in the environment of foals [44]–[46], making it challenging to control exposure to the pathogen without creating other possible sources of bias. Finally, we acknowledge that whole blood leukocytes were used for the purpose of this study. Thus, it is possible that differences in expression profiles were related to differences in subpopulations of leukocytes. Although this limitation would not have affected our within-age comparisons (where the distribution of cell subpopulations would have been the same for individual foals), it is relevant to our between age comparisons. We note, however, that our total leukocyte counts across age showed no significant differences among ages, and the ratio of neutrophils to lymphocytes was never less than 1.3 (unpublished data, not included in the manuscript). A tenable case can be made that the gene expression profile of those leukocytes in circulation at a given age better reflect the expected composite host response to infection than those of isolated subpopulations. Indeed, this was our rationale for examining total leukocytes in this study.

### Conclusions

We characterized the age-related gene expression profile of neonatal foals during the first 8 weeks of life associated with stimulation of peripheral blood leukocytes by *R. equi*. We also identified genes that were modulated temporally when blood leukocytes were stimulated with virulent *R. equi*. In this study we report the induction of innate immune response genes by stimulation of neonatal foal leukocytes with *R. equi*. Induction of such genes is likely critical in defense against *R. equi*, and lowered expression/repression of such genes in certain foals could render them highly susceptible to develop *R. equi* pneumonia once exposed to the pathogen. While it appears that foals of up to 8 weeks of age are able to initiate a protective defense and inflammatory response against the bacteria, relatively decreased expression or repression of various other immune-related genes points toward diminished immune responses closer to birth. The relatively reduced/repressed expression of such genes could also reflect immune-modulation of host responses by the pathogen for its survival. If foals generally become infected early in life, as supported by some research findings [7], [8], the genes and associated pathways identified by this study could be targeted for disease prevention or for developing novel therapeutic interventions. For example, enhanced MHC II/Th1-type immune responses might help protect foals against *R. equi* and other intracellular pathogens (e.g., *Salmonella*) during early life.

## Supporting Information

Figure S1 **Toll-like receptor signaling pathways.** Red arrows indicate the genes involved in TLR2 and TLR9 signaling pathways differentially expressed in *R. equi*-stimulated leukocytes compared to unstimulated. Illustration reproduced, courtesy of Cell Signaling Technology, Inc. (www.cellsignal.com).
(TIF)

Figure S2 **NF-kB activation cascade.** Red arrows indicate the genes involved in NF- **kB** activation that are differentially expressed in *R. equi*-stimulated leukocytes compared to unstimulated. Illustration reproduced, courtesy of Cell Signaling Technology, Inc. (www.cellsignal.com).
(TIF)

Table S1 **List of primers for real-time PCR validation.**
(DOCX)

Table S2 **Functional analysis of up-regulated genes in C1.**
(DOCX)

Table S3 **Functional analysis of up-regulated genes for C2.**
(DOCX)

Table S4 **Functional analysis of up-regulated genes in C3.**
(DOCX)

Table S5 **List of differentially expressed genes (pvalue <0.05 and fold-change cut off of 1.5) between the stimulated and the unstimulated leukocytes at Day 1.**
(DOCX)

Table S6 **List of differentially expressed genes (pvalue <0.05 and fold-change cut off of 1.5) between the stimulated and the unstimulated leukocytes at Week-2.**
(DOCX)

Table S7 **List of differentially expressed genes (pvalue <0.05 and fold-change cut off of 1.5) between the stimulated and the unstimulated leukocytes at Week-4.**
(DOCX)

Table S8 **List of differentially expressed genes (pvalue <0.05 and fold-change cut off of 1.5) between the stimulated and the unstimulated leukocytes at Week-8.**
(DOCX)

Table S9 **List of differentially expressed genes (pvalue <0.05 and fold-change cut off of 1.5) between the stimulated leukocytes at Week-2 compared to Day 1.**
(DOCX)

Table S10 **List of differentially expressed genes (pvalue <0.05 and fold-change cut off of 1.5) between the stimulated leukocytes at Week-4 compared to Day 1.**
(DOCX)

Table S11 **List of differentially expressed genes (pvalue <0.05 and fold-change cut off of 1.5) between the stimulated leukocytes at Week-8 compared to Day 1.**
(DOCX)

## References

[1] Meijer WG, Prescott JF (2004) *Rhodococcus equi* . Vet Res 35: 383–396. 15236672 10.1051/vetres:2004024

[2] Muscatello G, Leadon DP, Klayt M, Ocampo-Sosa A, Lewis DA, et al (2007) *Rhodococcus equi* infection in foals: the science of ‘rattles’. Equine Vet J 39: 470–478. 17910275 10.2746/042516407x209217

[3] Prescott JF (1991) *Rhodococcus equi*: an animal and human pathogen. Clin Microbiol Rev 4: 20–34. 2004346 10.1128/cmr.4.1.20 PMC358176

[4] Takai S (1997) Epidemiology of *Rhodococcus equi* infections: a review. Vet Microbiol 56: 167–176. 9226831 10.1016/s0378-1135(97)00085-0

[5] Giguere S, Cohen ND, Chaffin MK, Hines SA, Hondalus MK, et al (2011) *Rhodococcus equi*: clinical manifestations, virulence, and immunity. J Vet Intern Med 25: 1221–1230. 22092609 10.1111/j.1939-1676.2011.00804.x

[6] Giguere S, Cohen ND, Chaffin MK, Slovis NM, Hondalus MK, et al (2011) Diagnosis, treatment, control, and prevention of infections caused by *Rhodococcus equi* in foals. J Vet Intern Med 25: 1209–1220. 22092608 10.1111/j.1939-1676.2011.00835.x

[7] Chaffin MK, Cohen ND, Martens RJ (2008) Chemoprophylactic effects of azithromycin against *Rhodococcus equi*-induced pneumonia among foals at equine breeding farms with endemic infections. J Am Vet Med Assoc 232: 1035–1047. 18380623 10.2460/javma.232.7.1035

[8] Horowitz ML, Cohen ND, Takai S, Becu T, Chaffin MK, et al (2001) Application of Sartwell’s model (lognormal distribution of incubation periods) to age at onset and age at death of foals with *Rhodococcus equi* pneumonia as evidence of perinatal infection. J Vet Intern Med 15: 171–175. 11380023 10.1892/0891-6640(2001)015<0171:aosmld>2.3.co;2

[9] Breathnach CC, Sturgill-Wright T, Stiltner JL, Adams AA, Lunn DP, et al (2006) Foals are interferon gamma-deficient at birth. Vet Immunol Immunopathol 112: 199–209. 16621024 10.1016/j.vetimm.2006.02.010

[10] Darrah PA, Monaco MC, Jain S, Hondalus MK, Golenbock DT, et al (2004) Innate immune responses to *Rhodococcus equi* . J Immunol 173: 1914–1924. 15265925 10.4049/jimmunol.173.3.1914

[11] Liu T, Nerren J, Liu M, Martens R, Cohen N (2009) Basal and stimulus-induced cytokine expression is selectively impaired in peripheral blood mononuclear cells of newborn foals. Vaccine 27: 674–683. 19056444 10.1016/j.vaccine.2008.11.040

[12] Stears RL, Getts RC, Gullans SR (2000) A novel, sensitive detection system for high-density microarrays using dendrimer technology. Physiol Genomics 3: 93–99. 11015604 10.1152/physiolgenomics.2000.3.2.93

[13] Dobbin K, Shih JH, Simon R (2003) Statistical design of reverse dye microarrays. Bioinformatics 19: 803–810. 12724289 10.1093/bioinformatics/btg076

[14] Bright LA, Burgess SC, Chowdhary B, Swiderski CE, McCarthy FM (2009) Structural and functional-annotation of an equine whole genome oligoarray. BMC Bioinformatics 10 Suppl 11. 10.1186/1471-2105-10-S11-S8 PMC3226197 19811692

[15] Smyth GK (2004) Linear models and empirical bayes methods for assessing differential expression in microarray experiments. Stat Appl Genet Mol Biol 3. 10.2202/1544-6115.1027 16646809

[16] Smyth GK, Speed T (2003) Normalization of cDNA microarray data. Methods 31: 265–273. 14597310 10.1016/s1046-2023(03)00155-5

[17] Smyth GK, Speed T (2003) Normalization of cDNA microarray data. Methods 31: 265–273. 14597310 10.1016/s1046-2023(03)00155-5

[18] Benjamini Y, Hochberg Y (1995) Controlling the False Discovery Rate: A Practical and Powerful Approach to Multiple Testing. J R Stat Soc B 57: 289–300.

[19] Zhang B, Kirov S, Snoddy J (2005) WebGestalt: an integrated system for exploring gene sets in various biological contexts. Nucleic Acids Res 33: W741–748. 15980575 10.1093/nar/gki475 PMC1160236

[20] Duncan D, Prodduturi N, Zhang B (2010) WebGestalt2: an updated and expanded version of the Web-based Gene Set Analysis Toolkit. BMC Bioinformatics 11: 1–1.20043860

[21] Pfaffl MW (2001) A new mathematical model for relative quantification in real-time RT-PCR. Nucleic Acids Res 29. 10.1093/nar/29.9.e45 PMC55695 11328886

[22] Flaminio MJ, Nydam DV, Marquis H, Matychak MB, Giguere S (2009) Foal monocyte-derived dendritic cells become activated upon *Rhodococcus equi* infection. Clin Vaccine Immunol 16: 176–183. 19109450 10.1128/CVI.00336-08 PMC2643540

[23] Jacks S, Giguere S, Crawford PC, Castleman WL (2007) Experimental infection of neonatal foals with *Rhodococcus equi* triggers adult-like gamma interferon induction. Clin Vaccine Immunol 14: 669–677. 17409222 10.1128/CVI.00042-07 PMC1951072

[24] Ashida H, Mimuro H, Ogawa M, Kobayashi T, Sanada T, et al (2011) Cell death and infection: a double-edged sword for host and pathogen survival. Journal of Cell Biology 195: 931–942. 22123830 10.1083/jcb.201108081 PMC3241725

[25] Hartung T, Fennrich S, Fischer M, Montag-Lessing T, Wendel A (1998) Development and evaluation of a pyrogen test based on human whole blood. ALTEX 15: 9–10. 11178526

[26] Burger D, Dayer JM, Palmer G, Gabay C (2006) Is IL-1 a good therapeutic target in the treatment of arthritis? Best Pract Res Clin Rheumatol 20: 879–896. 16980212 10.1016/j.berh.2006.06.004

[27] Martens RJ, Cohen ND, Jones SL, Moore TA, Edwards JF (2005) Protective role of neutrophils in mice experimentally infected with *Rhodococcus equi* . Infect Immun 73: 7040–7042. 16177388 10.1128/IAI.73.10.7040-7042.2005 PMC1230988

[28] Perry AK, Chen G, Zheng D, Tang H, Cheng G (2005) The host type I interferon response to viral and bacterial infections. Cell Research 15: 407–422. 15987599 10.1038/sj.cr.7290309

[29] Schutyser E, Struyf S, Van Damme J (2003) The CC chemokine CCL20 and its receptor CCR6. Cytokine and Growth Factor Reviews 14: 409–426. 12948524 10.1016/s1359-6101(03)00049-2

[30] Lande R, Giacomini E, Grassi T, Remoli ME, Iona E, et al (2003) IFN-alpha beta released by Mycobacterium tuberculosis-infected human dendritic cells induces the expression of CXCL10: selective recruitment of NK and activated T cells. J Immunol 170: 1174–1182. 12538673 10.4049/jimmunol.170.3.1174

[31] Kanaly ST, Hines SA, Palmer GH (1993) Failure of pulmonary clearance of *Rhodococcus equi* infection in CD4+ T-lymphocyte-deficient transgenic mice. Infect Immun 61: 4929–4932. 8104903 10.1128/iai.61.11.4929-4932.1993 PMC281259

[32] Hardaker EL, Bacon AM, Carlson K, Roshak AK, Foley JJ, et al (2004) Regulation of TNF-alpha- and IFN-gamma-induced CXCL10 expression: participation of the airway smooth muscle in the pulmonary inflammatory response in chronic obstructive pulmonary disease. FASEB Journal 18: 191–193. 14597565 10.1096/fj.03-0170fje

[33] Boyd NK, Cohen ND, Lim WS, Martens RJ, Chaffin MK, et al (2003) Temporal changes in cytokine expression of foals during the first month of life. Vet Immunol Immunopathol 92: 75–85. 12628765 10.1016/s0165-2427(03)00021-7

[34] MacMicking JD (2004) IFN-inducible GTPases and immunity to intracellular pathogens. Trends Immunol 25: 601–609. 15489189 10.1016/j.it.2004.08.010

[35] Wang C, Morley SC, Donermeyer D, Peng I, Lee WP, et al (2010) Actin-bundling protein L-plastin regulates T cell activation. J Immunol 185: 7487–7497. 21076065 10.4049/jimmunol.1001424 PMC3027212

[36] Heller MC, Jackson KA, Watson JL (2010) Identification of immunologically relevant genes in mare and foal dendritic cells responding to infection by *Rhodococcus equi* . Vet Immunol Immunopathol 136: 144–150. 20334935 10.1016/j.vetimm.2010.02.016

[37] Sellon DC, Levine JF, Palmer K, Millikin E, Grindem C, et al (1997) Thrombocytosis in 24 horses (1989–1994). Journal of Veterinary Internal Medicine 11: 24–29. 9132480 10.1111/j.1939-1676.1997.tb00069.x

[38] Buyukasik Y, Soylu B, Soylu AR, Ozcebe OI, Canbakan S, et al (1998) In vivo platelet and T-lymphocyte activities during pulmonary tuberculosis. Eur Respir J 12: 1375–1379. 9877494 10.1183/09031936.98.12061375

[39] Smith R, 3rd, Chaffin MK, Cohen ND, Martens RJ (2002) Age-related changes in lymphocyte subsets of quarter horse foals. American Journal of Veterinary Research 63: 531–537. 11939315 10.2460/ajvr.2002.63.531

[40] Gercken J, Pryjma J, Ernst M, Flad HD (1994) Defective antigen presentation by *Mycobacterium tuberculosis*-infected monocytes. Infection and Immunity 62: 3472–3478. 8039918 10.1128/iai.62.8.3472-3478.1994 PMC302980

[41] Heller MC, Drew CP, Jackson KA, Griffey S, Watson JL (2010) A potential role for indoleamine 2,3-dioxygenase (IDO) in *Rhodococcus equi* infection. Veterinary Immunology and Immunopathology 138: 174–182. 20739070 10.1016/j.vetimm.2010.07.013

[42] Rivero-Lezcano OM, Gonzalez-Cortes C, Reyes-Ruvalcaba D, Diez-Tascon C (2010) CCL20 is overexpressed in Mycobacterium tuberculosis-infected monocytes and inhibits the production of reactive oxygen species (ROS). Clinical and Experimental Immunology 162: 289–297. 20819093 10.1111/j.1365-2249.2010.04168.x PMC2996596

[43] Bordin AI, Liu M, Nerren JR, Buntain SL, Brake CN, et al (2012) Neutrophil function of neonatal foals is enhanced in vitro by CpG oligodeoxynucleotide stimulation. Veterinary Immunology and Immunopathology 145: 290–297. 22197007 10.1016/j.vetimm.2011.11.012

[44] Cohen ND, Carter CN, Scott HM, Chaffin MK, Smith JL, et al (2008) Association of soil concentrations of *Rhodococcus equi* and incidence of pneumonia attributable to *Rhodococcus equi* in foals on farms in central Kentucky. Am J Vet Res 69: 385–395. 18312138 10.2460/ajvr.69.3.385

[45] Grimm MB, Cohen ND, Slovis NM, Mundy GD, Harrington JR, et al (2007) Evaluation of fecal samples from mares as a source of *Rhodococcus equi* for their foals by use of quantitative bacteriologic culture and colony immunoblot analyses. Am J Vet Res 68: 63–71. 17199420 10.2460/ajvr.68.1.63

[46] Muscatello G, Anderson GA, Gilkerson JR, Browning GF (2006) Associations between the ecology of virulent *Rhodococcus equi* and the epidemiology of *R. equi* pneumonia on Australian thoroughbred farms. Appl Environ Microbiol 72: 6152–6160. 16957241 10.1128/AEM.00495-06 PMC1563629

