# Supplementary figures and images for: Age-Related Changes following In Vitro Stimulation with Rhodococcus equi of Peripheral Blood Leukocytes from Neonatal Foals
Source: PLoS One. 2013 May 17;8(5):e62879. doi: 10.1371/journal.pone.0062879 (PMC3656898; doi:10.1371/journal.pone.0062879)

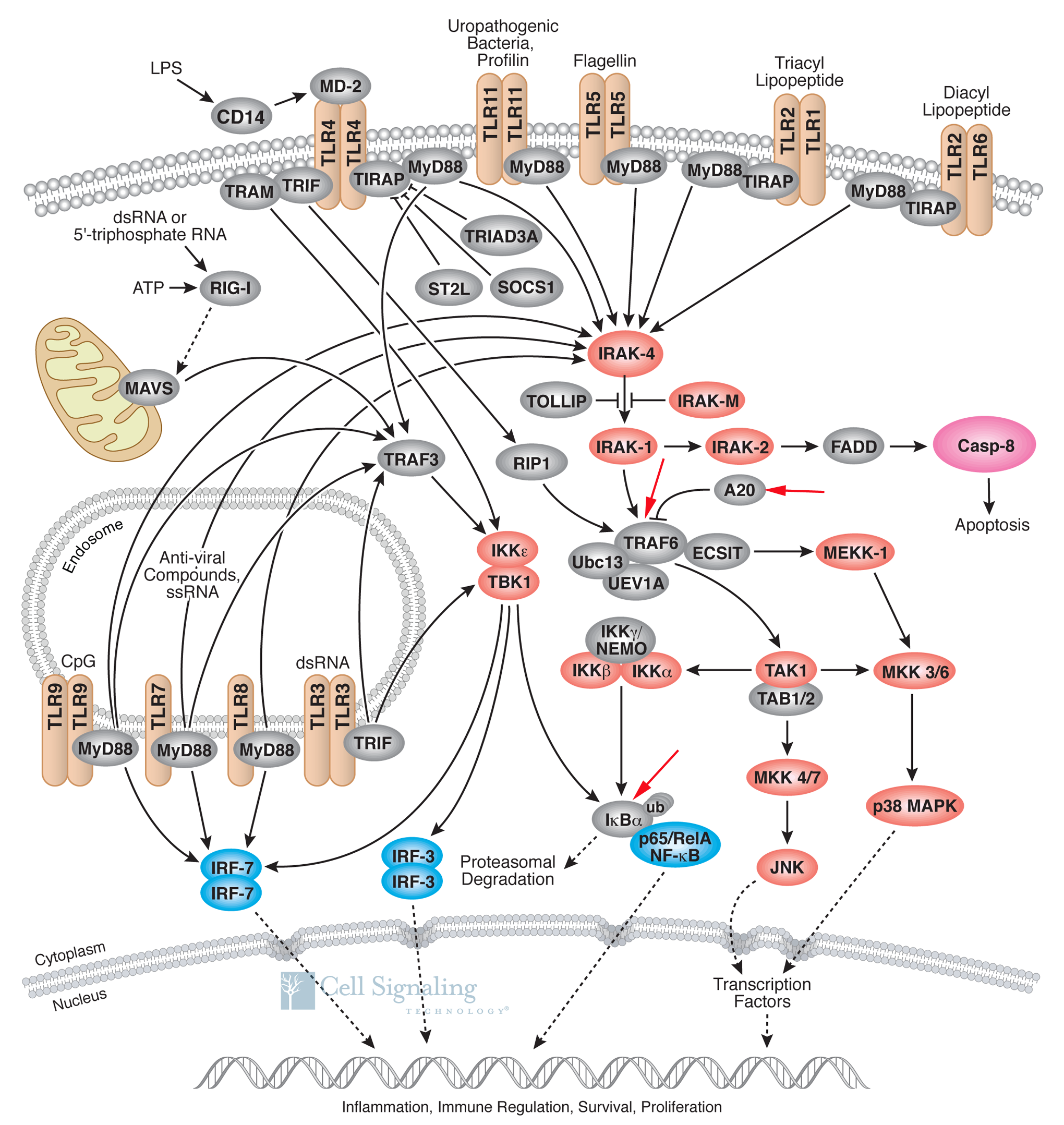

Supplement: Figure S1 — Toll-like receptor signaling pathways. Red arrows indicate the genes involved in TLR2 and TLR9 signaling pathways differentially expressed in R. equi-stimulated leukocytes compared to unstimulated. Illustration reproduced, courtesy of Cell Signaling Technology, Inc. (www.cellsignal.com). (TIF) [file pone.0062879.s001.tif]

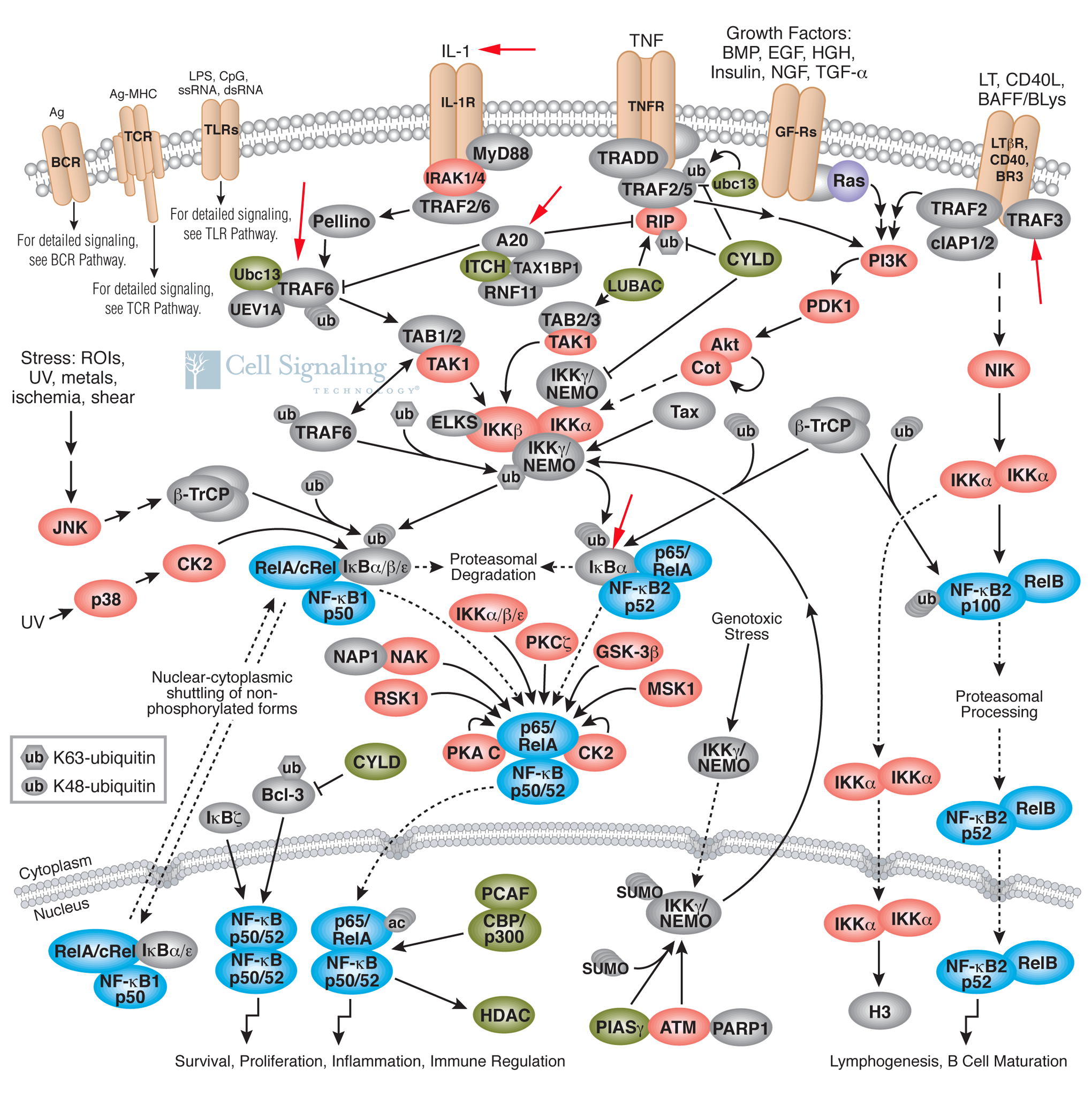

Supplement: Figure S2 — NF-kB activation cascade. Red arrows indicate the genes involved in NF- kB activation that are differentially expressed in R. equi-stimulated leukocytes compared to unstimulated. Illustration reproduced, courtesy of Cell Signaling Technology, Inc. (www.cellsignal.com). (TIF) [file pone.0062879.s002.tif]
