# Supplementary material for: Age-Related Changes following In Vitro Stimulation with Rhodococcus equi of Peripheral Blood Leukocytes from Neonatal Foals
Source: PLoS One. 2013 May 17;8(5):e62879. doi: 10.1371/journal.pone.0062879 (PMC3656898; doi:10.1371/journal.pone.0062879)
Supplement: Table S1 — List of primers for real-time PCR validation. (DOCX) [file pone.0062879.s003.docx]

**Table S1**

| Gene Symbol | Accession | Sequence |
| --- | --- | --- |
| TRAF3 | XM_001490000.1 | F: CTAAAGCTGCACCCTGACC |
|  |  | R: GTCCTCCACTGTCTTCACAAA |
| IL1B | XM_001495926.1 | F: CCGACACCAGTGACATGATGA |
|  |  | R: ATCCTCCTCAAAGAACAGGTCATTC |
| NFKBIA | XM_001491378 | F: GTCGCTCTTGTTGAAATGTG |
|  |  | R: CCTGGTAGGTGACTCTGTTGA |
| IFNG | NM_001081949 | F: GGCCTAACTCTCTCCGAAAC |
|  |  | R: TGGCAGTAATAGGTAGAAGAACC |
| ACTB | NM_001081838 | F: CCCAGATCATGTTTGAGACCT |
|  |  | R: CCTCGTAGATGGGCACAGT |
