# Supplementary material for: Age-Related Changes following In Vitro Stimulation with Rhodococcus equi of Peripheral Blood Leukocytes from Neonatal Foals
Source: PLoS One. 2013 May 17;8(5):e62879. doi: 10.1371/journal.pone.0062879 (PMC3656898; doi:10.1371/journal.pone.0062879)
Supplement: Table S2 — Functional analysis of up-regulated genes in C1. (DOCX) [file pone.0062879.s004.docx]

**Table S2a**

| Term | Genes | P-value |
| --- | --- | --- |
| GO:0002504~antigen processing and presentation of peptide or polysaccharide antigen via MHC class II | \| HLA-DQB1, HLA-DRA \| \| --- \| \|  \| | 0.02664453 |
| GO:0019538~protein metabolic process | RPS25, RPSA, EEF1A1, TTBK2, CXCR4, THBS1 | 0.059407109 |
| GO:0044260~cellular macromolecule metabolic process | RPS25, CRTC3, RPSA, EEF1A1, ZFP14, TTBK2, CXCR4, THBS1 | 0.084489257 |
| GO:0006952~defense response | RPS25, CRTC3, RPSA, EEF1A1, ZFP14, TTBK2, CXCR4, THBS1 | 0.087210365 |

**Table S2b**

| Term | Genes | P-value |
| --- | --- | --- |
| GO:0009308~amine metabolic process | 0.006314697 | CPT1B, MTHFD2L, AZIN1, B3GNT2, PLA2G5, CBS |
| GO:0006519~cellular amino acid and derivative metabolic process | 0.020021027 | CPT1B, MTHFD2L, AZIN1, PLA2G5, CBS |
| GO:0006082~organic acid metabolic process | 0.024210608 | ACADVL, CPT1B, MTHFD2L, PGM1, PLA2G5, CBS |
| GO:0042180~cellular ketone metabolic process | 0.025381787 | ACADVL, CPT1B, MTHFD2L, PGM1, PLA2G5, CBS |
| GO:0051649~establishment of localization in cell | 0.038408862 | FYB, CPT1B, TSPO, TRAK1, SDCBP, CLINT1, ARFGEF1 |
| GO:0046907~intracellular transport | 0.043784823 | FYB, CPT1B, TSPO, TRAK1, SDCBP, CLINT1 |
| GO:0006091~generation of precursor metabolites and energy | 0.065439973 | ATP6V1C1, ACADVL, GNPDA1, PGM1 |
| GO:0016042~lipid catabolic process | 0.093274145 | ACADVL, CPT1B, PLA2G5 |
| GO:0042439~ethanolamine and derivative metabolic process | 0.096331193 | CPT1B, PLA2G5 |
