# Supplementary material for: Age-Related Changes following In Vitro Stimulation with Rhodococcus equi of Peripheral Blood Leukocytes from Neonatal Foals
Source: PLoS One. 2013 May 17;8(5):e62879. doi: 10.1371/journal.pone.0062879 (PMC3656898; doi:10.1371/journal.pone.0062879)
Supplement: Table S3 — Functional analysis of up-regulated genes for C2. (DOCX) [file pone.0062879.s005.docx]

**Table S3a**

|  |  |  |
| --- | --- | --- |
| GO:0008104~protein localization | 0.054298499 | CBLB, CLIC5, VPS54, JAK2, TLK1, MAL, ERCC3 |
| GO:0046677~response to antibiotic | 0.07809548 | ALPL, JAK2 |
| GO:0051098~regulation of binding | 0.082262103 | PIM1, ZNF462, JAK2 |
| GO:0006811~ion transport | 0.08907577 | CLCA2, ATP6V0E1, KCNK7, CLIC5, CACNG1, ATP13A2 |

**Table S3b**

|  |  |  |
| --- | --- | --- |
| GO:0006810~transport | 0.03425892 | FYB, TSPO, ATP1B3, CACHD1, PLA2G10, ATP11B, NFKBIA, SNX3, KCNJ2, KCNK2, ARFGEF1, ATP6V1C1, PICALM, RAB18, LRMP, SDCBP, SCG5, CLINT1, PLA2G5 |
| GO:0051049~regulation of transport | 0.044044402 | TSPO, PLA2G10, SAA1, EDN2, NFKBIA, SCG5 |
| GO:0032370~positive regulation of lipid transport | 0.047962746 | PLA2G10, NFKBIA |
| GO:0046483~heterocycle metabolic process | 0.061427564 | ATP6V1C1, TSPO, MTHFD2L, ATP1B3, ATP11B |
| GO:0006897~endocytosis | 0.074805014 | PICALM, RAB18, SNX3, CLINT1 |
| GO:0010324~membrane invagination | 0.074805014 | PICALM, RAB18, SNX3, CLINT1 |
| GO:0051050~positive regulation of transport | 0.077209754 | PLA2G10, SAA1, EDN2, NFKBIA |
| GO:0051649~establishment of localization in cell | 0.083396521 | FYB, TSPO, NFKBIA, SDCBP, LRMP, SCG5, CLINT1, ARFGEF1 |
| GO:0007599~hemostasis | 0.083616771 | ANXA7, SAA1, ANXA5 |
| GO:0051047~positive regulation of secretion | 0.084943316 | PLA2G10, SAA1, EDN2 |
| GO:0010883~regulation of lipid storage | 0.089597899 | PLA2G10, NFKBIA |
| GO:0002682~regulation of immune system process | 0.092285153 | CD47, PLA2G10, EDN2, NFKBIA, ORM2 |
| GO:0060341~regulation of cellular localization | 0.098535349 | SAA1, EDN2, NFKBIA, SCG5 |
