# Supplementary material for: Age-Related Changes following In Vitro Stimulation with Rhodococcus equi of Peripheral Blood Leukocytes from Neonatal Foals
Source: PLoS One. 2013 May 17;8(5):e62879. doi: 10.1371/journal.pone.0062879 (PMC3656898; doi:10.1371/journal.pone.0062879)
Supplement: Table S4 — Functional analysis of up-regulated genes in C3. (DOCX) [file pone.0062879.s006.docx]

**Table S4a**

|  |  |  |
| --- | --- | --- |
| GO:0019538~protein metabolic process | RPSA, EEF1A1, RPL36A, PAIP1, SHFM1, DNAJC10, RPS2, RPL28, RPS25, TOR2A, MAP4K4, CASP4, RPL7, RPS3A, RPL31, RPLP1, RPL10, SPCS1, RPL37A, HSPA8 | 7.94E-06 |
| GO:0002504~antigen processing and presentation of peptide or polysaccharide antigen via MHC class II | HLA-DQB1, HLA-DRB1, HLA-DRA | 0.003112202 |
| GO:0010467~gene expression | EEF1A1, RPSA, RPL36A, HOXA11, PAIP1, RPS2, RPL28, RPS25, RPL7, RPL31, RPS3A, RPLP1, RPL10, RPL37A, SPCS1 | 0.010362081 |
| GO:0044260~cellular macromolecule metabolic process | RPSA, EEF1A1, RPL36A, PAIP1, HOXA11, SHFM1, DNAJC10, RPS2, RPL28, RPS25, TOR2A, MAP4K4, RPL7, ARRB2, RPS3A, RPL31, RPLP1, RPL10, SPCS1, RPL37A, HSPA8 | 0.013814143 |
| GO:0009059~macromolecule biosynthetic process | EEF1A1, RPSA, RPL36A, HOXA11, PAIP1, RPS2, RPL28, RPS25, RPL7, RPL31, RPS3A, RPLP1, RPL10, RPL37A | 0.016360863 |
| GO:0044249~cellular biosynthetic process | EEF1A1, RPSA, RPL36A, HOXA11, PAIP1, ASNSD1, RPS2, RPL28, RPS25, RPL7, RPL31, RPS3A, RPLP1, RPL10, RPL37A | 0.033528823 |

**Table S4b**

|  |  |  |
| --- | --- | --- |
| GO:0051649~establishment of localization in cell | 0.042680488 | FYB, TSPO, SCYL1, SDCBP, CLINT1, ARFGEF1, WIPI1 |
| GO:0046907~intracellular transport | 0.047946454 | FYB, TSPO, SCYL1, SDCBP, CLINT1, WIPI1 |
| GO:0048193~Golgi vesicle transport | 0.060227981 | SCYL1, CLINT1, WIPI1 |
| GO:0046483~heterocycle metabolic process | 0.080865953 | ATP6V1C1, TSPO, ATP1B3, ATP11B |
| GO:0006810~transport | 0.088269228 | FYB, TSPO, ATP1B3, ATP11B, OXTR, SNX3, ARFGEF1, WIPI1, ATP6V1C1, SCYL1, SDCBP, CLINT1, PLA2G5 |
| GO:0016192~vesicle-mediated transport | 0.096853663 | SCYL1, SNX3, CLINT1, ARFGEF1, WIPI1 |
| GO:0016042~lipid catabolic process | 0.09726011 | ACADVL, EHHADH, PLA2G5 |
