# Supplementary material for: Age-Related Changes following In Vitro Stimulation with Rhodococcus equi of Peripheral Blood Leukocytes from Neonatal Foals
Source: PLoS One. 2013 May 17;8(5):e62879. doi: 10.1371/journal.pone.0062879 (PMC3656898; doi:10.1371/journal.pone.0062879)
Supplement: Table S5 — List of differentially expressed genes (pvalue <0.05 and fold-change cut off of 1.5) between the stimulated and the unstimulated leukocytes at Day 1. (DOCX) [file pone.0062879.s007.docx]

**Table S5**

| **Gene Symbol** | **NCBI accession** | **RefSeq accession** | **Log fold change** | **P-value** |
| --- | --- | --- | --- | --- |
| AFF4 | XM_001504421 | XP_001504471 | 0.736771554 | 0.001231228 |
| ARMC8 | XM_001496956 | XP_001497006 | 0.993034946 | 0.000143926 |
| ATP6V1C1 | XM_001494101 | XP_001494151 | 1.61495745 | 0.012828436 |
| AZIN1 | XM_001493984 | XP_001494034 | 1.30074439 | 0.001350399 |
| AZIN1 | XR_035783 | NULL | 0.97456889 | 0.011838909 |
| B3GALT4 | XM_001497099 | XP_001497149 | 0.868094259 | 0.0004303 |
| B4GALT5 | XM_001501176 | XP_001501226 | 0.740378146 | 0.001318002 |
| BIRC3 | XM_001499875 | XP_001499925 | 1.925939744 | 0.005072062 |
| C17orf56 | XM_001489994 | XP_001490044 | 1.205522855 | 0.009628448 |
| CCL20 | XM_001496798 | NULL | 2.610358728 | 0.006660321 |
| CHIC1 | XM_001504969 | XP_001505019 | 0.656790617 | 6.01E-05 |
| CITED1 | XM_001488044 | XP_001488094 | 0.690298431 | 0.022558711 |
| CLINT1 | CX604855 | NULL | 1.222249813 | 0.018676155 |
| CXorf21 | XM_001502412 | NULL | 0.724386169 | 0.019306103 |
| CXXC5 | NULL | NULL | -0.622772728 | 0.017828615 |
| DDX58 | XM_001497845 | XP_001497895 | 1.012974707 | 0.028022378 |
| EDN2 | AB079136 | NP_001075292 | 0.71238449 | 0.025477548 |
| FAM21C | CX603317 | NULL | 2.12223529 | 0.002892481 |
| FAM3A | XM_001492203 | XP_001492253 | 0.920262899 | 0.006131427 |
| FLJ22662 | XM_001497121 | XP_001497171 | -0.69439459 | 0.006104455 |
| FPRL1 | XM_001497411 | XP_001497461 | 1.038335127 | 0.009498748 |
| GALNT2 | XM_001496209 | XP_001496259 | -1.29368847 | 0.049578246 |
| GNAI3 | XM_001494890 | XP_001494940 | 1.152377992 | 0.00116789 |
| GNB3 | XM_001497120 | XP_001497170 | 0.706700578 | 0.001334301 |
| GPR109A | CD469236 | NULL | 0.906632573 | 0.032062321 |
| GYG1 | XM_001491640 | XP_001491690 | -0.656642951 | 0.027410007 |
| HAGHL | XM_001497250 | XP_001497300 | 0.865290302 | 0.000618082 |
| HIP1R | XM_001492795 | XP_001492845 | 0.750215372 | 0.000570017 |
| HN1L | NULL | NULL | -1.234627804 | 0.025963621 |
| HNRNPA1 | XM_001501027 | XP_001501077 | -1.127468753 | 0.036793513 |
| IFNA5 | XM_001495486 | NULL | 1.112483298 | 0.038252084 |
| IL1A | NM_001082500 | NULL | 2.413131186 | 0.03380699 |
| IL1B | XM_001495926 | XP_001495976 | 1.490260424 | 0.02739791 |
| IL1RN | U92482 | NP_001075994 | 1.876892686 | 0.000360812 |
| INS-IGF2 | XM_001492829 | NULL | 0.718615748 | 0.009247854 |
| IVNS1ABP | CX604411 | NULL | -0.77874227 | 0.006571008 |
| KCNK2 | XM_001488153 | XP_001488203 | 0.904674402 | 0.032685262 |
| KCNK7 | XM_001493848 | XP_001493898 | -0.948840153 | 0.039837617 |
| KIAA2026 | DN508828 | NULL | 1.252853964 | 0.003279472 |
| KIAA2026 | NULL | NULL | 0.83003415 | 0.006222284 |
| KIF21A | XM_001500023 | XP_001500073 | 0.983154746 | 0.042016242 |
| KLRB1 | XM_001499255 | XP_001499305 | 0.783208614 | 0.039042291 |

**Table S5** Continued

| **Gene symbol** | **NCBI accession** | **RefSeq accession** | **Log fold change** | **P-value** |
| --- | --- | --- | --- | --- |
| LCP1 | CD466266 | NULL | -0.788551364 | 0.01608422 |
| LILRA6 | AB120413 | NP_001075993 | -0.712406497 | 0.011624887 |
| LILRB4 | XM_001489413 | XP_001489463 | -0.615096867 | 0.02846047 |
| LMBR1L | XM_001504157 | XP_001504207 | 0.602115766 | 0.018912226 |
| MAPK14 | XM_001494719 | XP_001494769 | -0.59840028 | 0.011840746 |
| MMP13 | AF034087 | NP_001075273 | 0.628858225 | 0.031911612 |
| MPP4 | XM_001496932 | XP_001496982 | 1.9042037 | 0.008789771 |
| MS4A6A | XM_001493201 | XP_001493251 | -0.660051914 | 0.01734874 |
| MTHFD2 | XM_001500723 | XP_001500773 | 1.056604193 | 0.016126532 |
| NFKBIA | NULL | NULL | 0.841142358 | 0.016476399 |
| NLRP13 | XM_001490754 | XP_001490804 | 0.623449799 | 0.031212763 |
| NUBP1 | XR_035869 | NULL | -0.660362741 | 0.042452626 |
| NULL | NULL | NULL | 3.650643824 | 0.001230412 |
| NULL | BI961791 | NULL | 1.733094978 | 0.020336907 |
| NULL | NULL | NULL | 1.614431528 | 0.003423183 |
| NULL | NULL | NULL | 1.422963358 | 0.002011361 |
| NULL | XM_001489363 | NULL | 1.402783642 | 0.005658842 |
| NULL | NULL | NULL | 1.398857319 | 0.010660813 |
| NULL | DN508878 | NULL | 1.392061079 | 0.0378562 |
| NULL | CD469517 | NULL | 1.383517701 | 0.00115628 |
| NULL | CX604543 | NULL | 1.374177108 | 0.015431937 |
| NULL | CD465425 | NULL | 1.273417274 | 0.000468737 |
| NULL | DN507020 | NULL | 1.231137808 | 0.000808902 |
| NULL | CD535494 | NULL | 1.150001754 | 0.000533264 |
| NULL | BM414612 | NULL | 1.10496954 | 0.021648089 |
| NULL | NULL | NULL | 1.024929833 | 0.000965244 |
| NULL | DN508987 | NULL | 0.965068028 | 0.018353876 |
| NULL | NULL | NULL | 0.924807265 | 0.017895581 |
| NULL | NULL | NULL | 0.756502529 | 0.027365545 |
| NULL | XM_001503112 | NULL | 0.75175176 | 0.014359124 |
| NULL | NULL | NULL | 0.740663541 | 0.000707521 |
| NULL | NULL | NULL | 0.685719513 | 0.047748735 |
| NULL | CX601512 | NULL | 0.658767096 | 0.047800289 |
| NULL | NULL | NULL | 0.647880706 | 0.012898365 |
| NULL | NULL | NULL | 0.592962906 | 0.000124773 |
| NULL | CX604697 | NULL | 0.58437 | 0.047801191 |
| NULL | XM_001497251 | NULL | -1.222264504 | 0.022010221 |
| NULL | XM_001497156 | NULL | -1.017644465 | 0.04531373 |

**Table S5** Continued

| **Gene symbol** | **NCBI accession** | **RefSeq accession** | **Log fold change** | **P-value** |
| --- | --- | --- | --- | --- |
| NULL | DN509725 | NULL | -0.940950373 | 0.030122187 |
| NULL | CX605267 | NULL | -0.881213651 | 0.047078064 |
| NULL | CX600791 | NULL | -0.846206416 | 0.032060274 |
| NULL | NULL | NULL | -0.690425434 | 0.003427915 |
| NULL | NULL | NULL | -0.683315393 | 0.003232457 |
| NULL | NULL | NULL | -0.659463765 | 0.028386418 |
| NULL | CD468881 | NULL | -0.65132791 | 0.037279346 |
| NULL | NULL | NULL | -0.639249664 | 0.031076657 |
| NULL | XM_001499111 | XP_001499161 | -0.583273337 | 0.035671404 |
| OLAH | XM_001498643 | XP_001498693 | -0.842955156 | 0.022441262 |
| OLR1 | XM_001493960 | XP_001494010 | 1.654290003 | 0.042997441 |
| OR2W3 | XM_001498405 | XP_001498455 | 1.280015075 | 7.45E-05 |
| OR8U8 | XM_001496341 | NULL | -0.606710763 | 0.040760083 |
| OSBPL11 | XM_001501564 | XP_001501614 | -0.621347908 | 0.017476482 |
| OXTR | XM_001491665 | XP_001491715 | 0.639764911 | 0.008698872 |
| PABPC4 | XM_001503450 | XP_001503500 | 0.822893601 | 0.009078976 |
| PIK3AP1 | XM_001500468 | XP_001500518 | 1.195804752 | 0.004431402 |
| PLSCR1 | XM_001492309 | XP_001492359 | -0.768157181 | 0.00405952 |
| PODXL | XM_001498373 | XP_001498423 | 1.236325872 | 0.000287089 |
| PRPF39 | XM_001493416 | NULL | 0.944144709 | 0.025222003 |
| PSMB5 | XM_001494488 | XP_001494538 | 0.640391481 | 0.044227039 |
| PTAFR | XM_001503995 | XP_001504045 | 0.684922323 | 0.002152635 |
| RAB6IP1 | XM_001500797 | XP_001500847 | 0.860114154 | 0.00686056 |
| RALGDS | NULL | NULL | 0.581840191 | 0.034669024 |
| RASGEF1B | NULL | NULL | 1.308715804 | 0.01677066 |
| RGS2 | XM_001490543 | XP_001490593 | -0.998595853 | 0.00797569 |
| SCUBE1 | XM_001500812 | XP_001500862 | -0.949584788 | 0.041363431 |
| SELP | CD467527 | NULL | -0.613675509 | 0.029230938 |
| SERPINB1 | M91161 | NP_001075416 | -0.718289355 | 0.022790827 |
| SERPINE2 | XM_001495938 | XP_001495988 | 0.866235669 | 0.043403297 |
| SLC22A6 | XM_001495190 | XP_001495240 | 0.581444932 | 0.014295459 |
| SMARCA4 | XM_001490624 | XP_001490674 | 0.797659347 | 0.000547984 |
| SUMO2 | XM_001499526 | XP_001499576 | -0.692012317 | 0.019498782 |
| TANK | XM_001493298 | XP_001493348 | 0.929247306 | 0.001159495 |
| TCTE3 | XM_001488610 | XP_001488660 | -0.888890706 | 0.017066746 |
| TFCP2 | XM_001504307 | NULL | 1.922040404 | 0.001358919 |
| TGM2 | XM_001499729 | NULL | 1.516040134 | 0.005775403 |
| BIRC3 | XM_001504414 | XP_001504464 | 0.595735418 | 0.024467972 |
| TRAF3 | XM_001490000 | XP_001490050 | 0.634627522 | 0.002496081 |

**Table S5** Continued

| **Gene symbol** | **NCBI accession** | **RefSeq accession** | **Log fold change** | **P-value** |
| --- | --- | --- | --- | --- |
| UBP1 | XM_001489950 | XP_001490000 | 1.157457981 | 0.035246983 |
| UQCRH | CX603785 | XP_001495144 | 0.775721189 | 0.040370337 |
| USP13 | XM_001496315 | XP_001496365 | 0.639778431 | 0.017319985 |
| ZNF462 | XM_001493275 | XP_001493325 | -0.81658804 | 0.037827707 |
| ZPBP2 | XM_001497917 | NULL | 0.625513646 | 0.003179897 |
