# Supplementary material for: Age-Related Changes following In Vitro Stimulation with Rhodococcus equi of Peripheral Blood Leukocytes from Neonatal Foals
Source: PLoS One. 2013 May 17;8(5):e62879. doi: 10.1371/journal.pone.0062879 (PMC3656898; doi:10.1371/journal.pone.0062879)
Supplement: Table S6 — List of differentially expressed genes (pvalue <0.05 and fold-change cut off of 1.5) between the stimulated and the unstimulated leukocytes at Week-2. (DOCX) [file pone.0062879.s008.docx]

**Table S6**

| **Gene symbol** | **NCBI accession** | **RefSeq accession** | **Log fold change** | **p-value** |
| --- | --- | --- | --- | --- |
| AACS | XM_001493527 | XP_001493577 | 0.697875092 | 0.007947887 |
| ALG8 | XM_001492875 | XP_001492925 | -0.728984573 | 0.048304908 |
| ALPL | XM_001504312 | XP_001504362 | -0.686710111 | 0.025516009 |
| ATP11B | XM_001496792 | XP_001496842 | 0.665612005 | 0.010133154 |
| ATP6V1C1 | XM_001494101 | XP_001494151 | 0.975560628 | 0.019712683 |
| BIRC3 | XM_001499875 | XP_001499925 | 1.615070966 | 7.11E-05 |
| BTN2A1 | XM_001492502 | XP_001492552 | 1.206141566 | 0.018929092 |
| C11orf49 | XR_036183 | NULL | 0.730436448 | 0.027136315 |
| C11orf49 | XR_035773 | NULL | 0.642278149 | 0.000285858 |
| C18orf22 | XM_001496059 | XP_001496109 | 0.80602411 | 0.003109621 |
| C21orf91 | XM_001500343 | XP_001500393 | 0.595403623 | 0.020166088 |
| CABP2 | XM_001498045 | NULL | 0.718691313 | 0.045264044 |
| CAST | XM_001503694 | XP_001503744 | -1.115287256 | 0.040730863 |
| CCDC100 | XM_001504520 | XP_001504570 | 0.803480114 | 0.000671344 |
| CLCN6 | XM_001491741 | XP_001491791 | -0.948193616 | 0.035580709 |
| CLDN14 | XM_001495856 | XP_001495906 | 0.912178907 | 0.040836892 |
| CUL3 | XM_001493366 | XP_001493416 | 0.709298728 | 0.021485139 |
| CXCL10 | XM_001490541 | XP_001490591 | 1.171016563 | 0.01448367 |
| CXCL3 | XM_001489197 | XP_001489247 | 0.654880213 | 0.013169831 |
| DDX58 | XM_001497845 | XP_001497895 | 1.353450442 | 0.000135334 |
| EDN2 | AB079136 | NP_001075292 | 0.729300071 | 0.034286293 |
| ELL2 | XR_036497 | NULL | 0.766178866 | 0.00996606 |
| FAM21C | CX603317 | NULL | 0.681328551 | 0.007521442 |
| FAM89A | NULL | NULL | -1.392747977 | 0.015022384 |
| GCN5L2 | XM_001495089 | XP_001495139 | -0.778986964 | 0.043977187 |
| GLB1L | XM_001493157 | XP_001493207 | -0.907075214 | 0.017892875 |
| GPR31 | XM_001489448 | XP_001489498 | 0.75204151 | 0.002582712 |
| GRB7 | XM_001501037 | XP_001501087 | 0.788591024 | 0.030267131 |
| HLF | XM_001500211 | XP_001500261 | 0.631119475 | 0.012749438 |
| ICAM2 | XM_001495313 | XP_001495363 | -0.946848255 | 0.034909114 |
| ID3 | XM_001504221 | XP_001504271 | -0.978905753 | 0.048206851 |
| IL1A | NM_001082500 | NULL | 2.585107345 | 0.00267813 |
| IL1B | XM_001495926 | XP_001495976 | 1.117415389 | 0.003005676 |
| IL1RN | U92482 | NP_001075994 | 1.735601259 | 0.008432987 |
| IMPG2 | XM_001503461 | XP_001503511 | 0.580153251 | 0.014373984 |
| INDO | XM_001490681 | XP_001490731 | 0.699291416 | 0.020064572 |
| KCNA3 | XM_001494050 | XP_001494100 | -0.781951055 | 0.021939954 |
| KCNJ2 | XM_001498612 | XP_001498662 | 1.146143623 | 0.009018466 |
| KIF21A | XM_001500023 | XP_001500073 | 0.981764562 | 0.006683527 |

**Table S6** Continued

| **Gene symbol** | **NCBI accession** | **RefSeq accession** | **Log fold change** | **P-value** |
| --- | --- | --- | --- | --- |
| KLHL20 | XM_001493014 | XP_001493064 | 0.698686792 | 0.004861024 |
| LIG4 | XM_001493890 | XP_001493940 | -0.795900702 | 0.04039123 |
| LMBR1L | XM_001504157 | XP_001504207 | 0.664735066 | 0.00043301 |
| LOC388630 | CX592898 | NULL | -0.630465559 | 0.046103315 |
| LOC728772 | XM_001491967 | XP_001492017 | 0.853607511 | 0.032762529 |
| LOC730422 | DN507079 | NP_001108413 | 2.084691798 | 0.000570065 |
| MCM6 | XM_001489698 | XP_001489748 | -1.193710812 | 0.044163362 |
| MEF2A | CD464185 | NULL | 0.640787956 | 0.048692709 |
| MEIS2 | XM_001503626 | XP_001503676 | -0.612615011 | 0.0420056 |
| NFKBIA | NULL | NULL | 0.76992866 | 0.012265461 |
| NR2F6 | XM_001499719 | XP_001499769 | -1.301052643 | 0.042924624 |
| NT5C2 | XM_001499520 | XP_001499570 | 1.018978363 | 0.033684678 |
| NULL | CD470350 | NULL | 1.746563419 | 0.001395949 |
| NULL | XM_001489363 | NULL | 1.665159892 | 5.30E-06 |
| NULL | CD469517 | NULL | 1.555468456 | 0.000114922 |
| NULL | DN508878 | NULL | 1.355154756 | 0.00526598 |
| NULL | BI961791 | NULL | 1.226659197 | 0.001107059 |
| NULL | CD469043 | NULL | 1.223220171 | 0.00356743 |
| NULL | CD465425 | NULL | 1.170032197 | 0.006003242 |
| NULL | BM414612 | NULL | 1.069382494 | 0.004701095 |
| NULL | DN508987 | NULL | 0.948631459 | 0.007527228 |
| NULL | CD465947 | NULL | 0.873054658 | 0.000692472 |
| NULL | CX604697 | NULL | 0.73798422 | 0.009839182 |
| NULL | CD536657 | NULL | 0.659792997 | 0.03513463 |
| NULL | BM734930 | NULL | 0.629887182 | 0.034901723 |
| NULL | CX602249 | NULL | 0.617207288 | 0.041069387 |
| NULL | CX604543 | NULL | 0.607069351 | 0.02211025 |
| NULL | CX606039 | NULL | 0.590691432 | 0.017643889 |
| NULL | XM_001499351 | NULL | -1.034739544 | 0.048989989 |
| NULL | CX604033 | NULL | -0.875177731 | 0.030203368 |
| NULL | DN509057 | NULL | -0.743127321 | 0.013027874 |
| NULL | CD467035 | NULL | -0.601842855 | 0.018924587 |
| OR13D1 | XM_001493177 | XP_001493227 | 0.596492155 | 0.038405249 |
| OR2W3 | XM_001498405 | XP_001498455 | 0.686014534 | 0.001182582 |
| OR7A5 | XM_001496119 | NULL | -1.411614429 | 0.041144358 |
| OR7E24 | XM_001493162 | NULL | 0.799036252 | 0.005243035 |
| OR8U8 | XM_001496341 | NULL | -1.53019112 | 0.004005738 |
| PICALM | XM_001490412 | XP_001490462 | 0.647383297 | 0.010620606 |
| PIK3AP1 | XM_001500468 | XP_001500518 | 0.893155484 | 0.003328656 |
| PLD5 | XM_001492999 | XP_001493049 | -0.659744978 | 0.031305064 |
| PLEK | XM_001492113 | XP_001492163 | 1.229628257 | 0.02832517 |

**Table S6** Continued

| **Gene symbol** | **NCBI accession** | **RefSeq accession** | **Log fold change** | **P-value** |
| --- | --- | --- | --- | --- |
| PNRC2 | XM_001504200 | NULL | 1.053665318 | 0.006980435 |
| PODXL | XM_001498373 | XP_001498423 | 0.687422164 | 0.002950615 |
| PRRC1 | XM_001504480 | XP_001504530 | -1.076634396 | 0.041794071 |
| PSCDBP | XM_001491278 | XP_001491328 | 1.272818912 | 0.000351192 |
| PTAFR | XM_001503995 | XP_001504045 | 0.822485473 | 0.007125783 |
| RASGEF1B | NULL | NULL | 0.848567319 | 0.012791089 |
| RETN | XM_001497441 | XP_001497491 | 0.580805519 | 0.030795519 |
| RGS2 | XM_001490543 | XP_001490593 | -0.658435263 | 0.004267861 |
| RNF19A | XM_001492262 | XP_001492312 | 0.930427638 | 0.001547354 |
| RNF24 | XM_001496498 | NULL | 1.927467483 | 0.048862351 |
| RPAP2 | XM_001492375 | XP_001492425 | -1.173849673 | 0.02198338 |
| SHARPIN | XM_001495880 | NULL | 0.746428938 | 0.015341767 |
| SLC35D2 | XM_001494155 | XP_001494205 | -0.940151368 | 0.023497194 |
| SMARCA4 | XM_001490624 | XP_001490674 | 0.96693218 | 0.000817632 |
| SNX10 | CD467781 | NULL | 0.973375468 | 0.046955258 |
| SOD2 | AB001693 | NP_001075986 | 1.64600987 | 0.003318969 |
| SPATA4 | XM_001493009 | XP_001493059 | -0.740639785 | 0.00537563 |
| STAMBPL1 | XM_001503046 | XP_001503096 | 0.966996845 | 0.035484165 |
| TFCP2 | XM_001504307 | NULL | 1.31403059 | 0.000400086 |
| TMLHE | XM_001498280 | XP_001498330 | -0.667880002 | 0.003929976 |
| TPR | XM_001487879 | NULL | 0.615968645 | 0.020129579 |
| TREM1 | XM_001500981 | XP_001501031 | 0.618781248 | 0.013385432 |
| UBP1 | XM_001489950 | XP_001490000 | 0.992715894 | 0.000908657 |
| USP13 | XM_001496315 | XP_001496365 | 0.664360613 | 0.004791727 |
| WDR51A | XR_036062 | NULL | 0.701553019 | 0.048494841 |
| YTHDF1 | XM_001492553 | XP_001492603 | 0.788093149 | 0.013563092 |
| ZNF462 | XM_001493275 | XP_001493325 | -1.309843924 | 0.040337679 |
| ZNF784 | XM_001490354 | XP_001490404 | 0.960078462 | 0.000240908 |
| ZSWIM6 | XM_001492480 | XP_001492530 | -0.635315737 | 0.0319159 |
