# Supplementary material for: Age-Related Changes following In Vitro Stimulation with Rhodococcus equi of Peripheral Blood Leukocytes from Neonatal Foals
Source: PLoS One. 2013 May 17;8(5):e62879. doi: 10.1371/journal.pone.0062879 (PMC3656898; doi:10.1371/journal.pone.0062879)
Supplement: Table S7 — List of differentially expressed genes (pvalue <0.05 and fold-change cut off of 1.5) between the stimulated and the unstimulated leukocytes at Week-4. (DOCX) [file pone.0062879.s009.docx]

**Table S7**

| **Gene Symbol** | **NCBI accession** | **RefSeq accession** | **Log fold change** | **P-value** |
| --- | --- | --- | --- | --- |
| ACTR2 | XM_001494389 | XP_001494439 | -1.442565254 | 0.003314642 |
| ADA | XM_001500443 | XP_001500493 | -0.875944536 | 0.024130993 |
| ARHGDIB | XM_001501778 | XP_001501828 | -0.637499858 | 0.038167737 |
| ARPC4 | CD528440 | NULL | -0.920637046 | 0.033625217 |
| BIRC3 | XM_001499875 | XP_001499925 | 1.280228588 | 0.006797131 |
| C14orf104 | XM_001496267 | XP_001496317 | 1.746290565 | 0.036210752 |
| C5orf36 | XM_001503712 | XP_001503762 | -0.603469059 | 0.044885869 |
| CXCL2 | AF053497 | NULL | 0.740888242 | 0.029366444 |
| CYP26A1 | XM_001502498 | XP_001502548 | -0.644005452 | 0.012248082 |
| DDX58 | XM_001497845 | XP_001497895 | 0.849356831 | 0.049516409 |
| EDEM3 | XM_001490885 | XP_001490935 | -0.944580951 | 0.031360262 |
| EDN2 | AB079136 | NP_001075292 | 0.607993768 | 0.025940492 |
| EIF4A1 | XM_001504774 | XP_001504824 | 1.071830095 | 0.048047913 |
| FGL2 | XM_001488486 | XP_001488536 | -0.66421589 | 0.03392253 |
| GABRB3 | XM_001493048 | XP_001493098 | -0.587994546 | 0.035456941 |
| IL1A | NM_001082500 | NULL | 2.572395297 | 0.000756361 |
| IL1B | XM_001495926 | XP_001495976 | 0.951317235 | 0.044939967 |
| IL1RN | U92482 | NP_001075994 | 1.729878284 | 0.027841748 |
| KCNJ10 | XM_001491211 | XP_001491261 | 0.639116173 | 0.001835555 |
| KCNJ2 | XM_001498612 | XP_001498662 | 0.652178111 | 0.015169015 |
| KIAA1434 | CD466056 | NULL | -0.652669269 | 0.043516761 |
| KIF21A | XM_001500023 | XP_001500073 | 0.780286691 | 0.014437963 |
| LILRB4 | CD467691 | NULL | 0.7487236 | 0.002005286 |
| LOC730796 | XM_001497173 | XP_001497223 | -1.864425085 | 0.015808519 |
| LYPLA1 | NULL | NULL | -0.581273371 | 0.030319939 |
| LYZL6 | XM_001494897 | XP_001494947 | -0.730179609 | 0.020052288 |
| MEF2A | CD464185 | NULL | 0.899356423 | 0.025180504 |
| NULL | CX605267 | NULL | 2.025224021 | 0.014351557 |
| NULL | XM_001499351 | NULL | 1.81497192 | 0.032856806 |
| NULL | BI961791 | NULL | 1.594257497 | 0.007960216 |
| NULL | BM414612 | NULL | 1.075807382 | 0.017516782 |
| NULL | CD469517 | NULL | 0.947058826 | 0.002164067 |
| NULL | CX602603 | NULL | 0.589551174 | 0.028049872 |
| NULL | CD536079 | NULL | -0.896364101 | 0.045862387 |
| NULL | CD465841 | NULL | -0.849233215 | 0.012961625 |
| NULL | CD467057 | NULL | -0.795731345 | 0.026775678 |
| NULL | CD528135 | NULL | -0.598270955 | 0.007291102 |
| OPA3 | DN509316 | NULL | -1.044739656 | 0.03591177 |
| OR1A1 | XM_001502552 | XP_001502602 | 0.592219414 | 0.007588206 |
| PLAU | XM_001502951 | XP_001503001 | 0.704530429 | 0.022173188 |
| PLEK | XM_001492113 | XP_001492163 | 1.102544441 | 0.013744419 |

**Table S7** Continued

| **Gene symbol** | **NCBI accession** | **RefSeq accession** | **Log fold change** | **P-value** |
| --- | --- | --- | --- | --- |
| PODXL | XM_001498373 | XP_001498423 | 0.613394511 | 0.013782635 |
| POLR1D | XM_001493249 | NULL | -1.2136447 | 0.023244224 |
| POLR1E | XM_001504281 | XP_001504331 | -0.862395043 | 0.025176069 |
| PSCDBP | XM_001491278 | XP_001491328 | 0.627090082 | 0.031605316 |
| RGS2 | XM_001490543 | XP_001490593 | -0.779635039 | 0.003267522 |
| RPS25 | XM_001503063 | XP_001503113 | -0.778312468 | 0.027655033 |
| SCG5 | XM_001501620 | XP_001501670 | -1.140674466 | 0.010563301 |
| SCYL1 | CX602064 | NULL | -0.780474666 | 0.044981942 |
| SELL | XM_001491555 | XP_001491605 | -0.648639128 | 0.023712227 |
| TFCP2 | XM_001504307 | NULL | 0.982984015 | 0.020718816 |
| TGM2 | XM_001499729 | NULL | -1.223681514 | 0.04554654 |
| TNNT3 | XM_001492908 | NULL | 0.625029157 | 0.015217817 |
| TREM1 | XM_001500981 | XP_001501031 | 0.69132632 | 0.016773534 |
| TSC22D3 | XM_001491157 | XP_001491207 | -0.624588592 | 0.01369984 |
| TTBK2 | NULL | NULL | -0.822268707 | 0.046272344 |
| USP13 | XM_001496315 | XP_001496365 | 0.630416799 | 0.003463127 |
| VIM | CX604176 | NULL | -0.756968459 | 0.036629821 |
| ZC3HAV1 | XM_001499163 | NULL | -0.613778865 | 0.038877421 |
| ZNF211 | XM_001494387 | XP_001494437 | -1.043226236 | 0.00886443 |
| ZNF407 | CX599900 | NULL | -1.052235826 | 0.041966686 |
| ZNF407 | NULL | NULL | -0.765018041 | 0.020102759 |
