# Supplementary material for: Age-Related Changes following In Vitro Stimulation with Rhodococcus equi of Peripheral Blood Leukocytes from Neonatal Foals
Source: PLoS One. 2013 May 17;8(5):e62879. doi: 10.1371/journal.pone.0062879 (PMC3656898; doi:10.1371/journal.pone.0062879)
Supplement: Table S8 — List of differentially expressed genes (pvalue <0.05 and fold-change cut off of 1.5) between the stimulated and the unstimulated leukocytes at Week-8. (DOCX) [file pone.0062879.s010.docx]

**Table S8**

| **Gene Symbol** | **NCBI accession** | **RefSeq accession** | **Log fold change** | **P-value** |
| --- | --- | --- | --- | --- |
| AK3 | DN510426 | NULL | -0.661739522 | 0.037256069 |
| ARMC8 | XM_001496956 | XP_001497006 | 0.846242545 | 0.000494223 |
| ARRDC3 | XM_001504616 | XP_001504666 | -0.819339052 | 0.024527401 |
| BIRC3 | XM_001499875 | XP_001499925 | 2.264968369 | 9.56E-05 |
| C11orf49 | XM_001494248 | XP_001494298 | 0.763452177 | 0.023052929 |
| C11orf49 | XR_036183 | NULL | 0.663628353 | 0.012780599 |
| C11orf49 | XR_036493 | NULL | 0.656792724 | 0.03831742 |
| C18orf22 | XM_001496059 | XP_001496109 | 1.328615535 | 0.001417922 |
| CCL20 | XM_001496798 | NULL | 1.107191516 | 0.006865372 |
| CFDP1 | XM_001498771 | XP_001498821 | 0.773997841 | 0.009567496 |
| CHCHD2 | XM_001499574 | XP_001499624 | -0.591928204 | 0.003264909 |
| CSNK1G3 | XM_001504513 | NULL | -0.598403293 | 0.020198351 |
| CUL3 | XM_001493366 | XP_001493416 | 0.979939517 | 0.008525381 |
| CXCL10 | XM_001490541 | XP_001490591 | 1.575531659 | 0.000560746 |
| CXCL2 | AF053497 | NULL | 0.917729157 | 0.002791755 |
| DCTN3 | DN509108 | XP_001503748 | -0.584589224 | 0.040841778 |
| DDX58 | XM_001497845 | XP_001497895 | 1.792685698 | 4.00E-06 |
| EDEM3 | XM_001490885 | XP_001490935 | -0.746385317 | 0.0292086 |
| EDN2 | AB079136 | NP_001075292 | 0.870137472 | 0.005503041 |
| FAM21C | CX603317 | NULL | 1.094500398 | 0.008875332 |
| GBP1 | XM_001494991 | XP_001495041 | 1.161217967 | 0.021352266 |
| GDI2 | XM_001500009 | XP_001500059 | -0.623018754 | 0.043096298 |
| GPR109A | CD469236 | NULL | 0.834857357 | 0.006961215 |
| GPR31 | XM_001489448 | XP_001489498 | 0.717358629 | 0.009769871 |
| GPR84 | XM_001504570 | XP_001504620 | 1.164376829 | 6.80E-05 |
| HEXIM2 | XM_001495090 | XP_001495140 | -1.014570409 | 0.021517129 |
| HNRNPU | CD528363 | NULL | -0.858937088 | 0.020943577 |
| HSPE1 | NULL | NULL | -1.329874116 | 0.010472361 |
| IFNA5 | XM_001495486 | NULL | 0.645616128 | 0.00248139 |
| IL1A | NM_001082500 | NULL | 3.829758886 | 1.78E-08 |
| IL1B | XM_001495926 | XP_001495976 | 2.162329197 | 0.0020009 |
| IL1RN | U92482 | NP_001075994 | 2.473567181 | 0.000714063 |
| INDO | XM_001490681 | XP_001490731 | 1.815170687 | 5.71E-05 |
| INS-IGF2 | XM_001492829 | NULL | 0.752345604 | 0.000182068 |
| IVNS1ABP | CX604411 | NULL | -0.926549023 | 0.002796688 |
| KCNJ2 | XM_001498612 | XP_001498662 | 1.27988629 | 0.001459713 |
| KIAA1434 | CD466056 | NULL | -0.79780471 | 0.007438089 |
| KIF21A | XM_001500023 | XP_001500073 | 1.268932109 | 0.000251705 |
| KLRB1 | XM_001499255 | XP_001499305 | 0.611917454 | 0.007104291 |
| KMO | XM_001492701 | XP_001492751 | 0.617044902 | 0.005072262 |
| LILRA6 | AB120413 | NP_001075993 | -0.698036649 | 0.004198966 |
| LILRB4 | XM_001489413 | XP_001489463 | -0.624058776 | 0.014811842 |

**Table S8** Continued

| **Gene Symbol** | | **NCBI accession** | **RefSeq accession** | **Log fold change** | **P-value** |
| --- | --- | --- | --- | --- | --- |
| LMBR1L | XM_001504157 | | XP_001504207 | 0.89806684 | 0.001025308 |
| LOC730422 | DN507079 | | NP_001108413 | 2.797778782 | 1.70E-05 |
| LOC730803 | CD471754 | | NULL | 0.741306132 | 0.000501277 |
| MPP4 | XM_001496932 | | XP_001496982 | 1.169375876 | 0.003034343 |
| NDUFA10 | XM_001500579 | | XP_001500629 | 1.46144557 | 0.035064978 |
| NFKBIA | NULL | | NULL | 0.909828808 | 0.001027568 |
| NPBWR2 | XM_001495569 | | NULL | 0.752061566 | 0.000326237 |
| NULL | CD469043 | | NULL | 1.927062928 | 0.004635845 |
| NULL | XM_001489363 | | NULL | 1.788861079 | 0.000157873 |
| NULL | DN508878 | | NULL | 1.788274041 | 7.03E-07 |
| NULL | CD470350 | | NULL | 1.753720715 | 0.004053683 |
| NULL | BI961791 | | NULL | 1.65394905 | 0.006544702 |
| NULL | CD469517 | | NULL | 1.522912202 | 2.92E-05 |
| NULL | BM414612 | | NULL | 1.146132093 | 0.007689025 |
| NULL | DN508987 | | NULL | 1.113626273 | 0.00016679 |
| NULL | CD465425 | | NULL | 1.036822143 | 0.004148771 |
| NULL | BI961659 | | NULL | 1.018131402 | 0.014146126 |
| NULL | CX604697 | | NULL | 0.962484971 | 0.000347426 |
| NULL | CD536657 | | NULL | 0.947982933 | 0.010354492 |
| NULL | DN507662 | | NULL | 0.875493921 | 0.014686101 |
| NULL | CX604543 | | NULL | 0.867957213 | 0.000213978 |
| NULL | CD467650 | | NULL | 0.759172053 | 0.038807186 |
| NULL | DN509862 | | NULL | 0.672955815 | 0.030058649 |
| NULL | CD470694 | | NULL | 0.594570216 | 0.00690229 |
| NULL | CX604033 | | NULL | -0.946244491 | 0.01018918 |
| NULL | CX601537 | | NULL | -0.740174279 | 0.018614933 |
| NULL | CX599471 | | NULL | -0.681775599 | 0.034322327 |
| NXF1 | AB302133 | | NP_001091076 | -0.611574648 | 0.047492306 |
| OLR1 | XM_001493960 | | XP_001494010 | 1.328882722 | 0.036496251 |
| OPA3 | DN509316 | | NULL | -0.863751882 | 0.005823285 |
| PGM1 | XM_001499673 | | XP_001499723 | 0.698336921 | 0.001334788 |
| PIK3AP1 | XM_001500468 | | XP_001500518 | 0.985168148 | 0.009981568 |
| PLAU | XM_001502951 | | XP_001503001 | 0.619602346 | 0.013212908 |
| PLEK | XM_001492113 | | XP_001492163 | 1.760980001 | 0.000304627 |
| PODXL | XM_001498373 | | XP_001498423 | 1.099337807 | 0.004288463 |
| PPFIA4 | XM_001495820 | | XP_001495870 | -0.708426479 | 0.048413006 |
| PSCDBP | XM_001491278 | | XP_001491328 | 0.806746862 | 0.005992711 |
| PSMB5 | XM_001494488 | | XP_001494538 | 0.637221712 | 0.002647985 |
| PTAFR | XM_001503995 | | XP_001504045 | 0.777576077 | 0.001461637 |
| RASGEF1B | NULL | | NULL | 0.712141042 | 0.007503619 |

**Table S8** Continued

| **Gene Symbol** | **NCBI accession** | **RefSeq accession** | **Log fold change** | **P-value** |
| --- | --- | --- | --- | --- |
| RGS2 | XM_001490543 | XP_001490593 | -0.94991284 | 0.000494958 |
| RHOG | XM_001496655 | XP_001496705 | 0.734130137 | 0.01977316 |
| RNASET2 | XM_001489592 | XP_001489642 | -0.713822838 | 0.02170683 |
| RNF19A | XM_001492262 | XP_001492312 | 0.864644553 | 0.029098453 |
| RSPH3 | XM_001491976 | XP_001492026 | 1.700046358 | 0.023920608 |
| SELL | XM_001491555 | XP_001491605 | -0.948017951 | 0.015346515 |
| SHPRH | XM_001502347 | XP_001502397 | -0.707018169 | 0.023369506 |
| SLC15A2 | XM_001500375 | XP_001500425 | 0.644046096 | 0.000764652 |
| SLC39A13 | XM_001491132 | XP_001491182 | -0.774930279 | 0.029339313 |
| SMARCA4 | XM_001490624 | XP_001490674 | 0.942879067 | 0.005320191 |
| SNX10 | CD467781 | NULL | 1.380222938 | 0.006243751 |
| SOD2 | AB001693 | NP_001075986 | 1.759764413 | 0.009343792 |
| TANK | XM_001493298 | XP_001493348 | 1.109704488 | 0.003522222 |
| TFCP2 | XM_001504307 | NULL | 2.056293477 | 4.33E-06 |
| TPI1 | XM_001497472 | XP_001497522 | 0.597111927 | 0.003084807 |
| TRAF6 | NULL | NULL | -0.726515334 | 0.013804121 |
| UBP1 | XM_001489950 | XP_001490000 | 0.823570172 | 0.015037027 |
| UMPS | XM_001500039 | XP_001500089 | 0.590924746 | 0.010610914 |
| USP13 | XM_001496315 | XP_001496365 | 0.589099914 | 0.006317992 |
| VAPB | XM_001490076 | XP_001490126 | 0.609593867 | 0.000700642 |
| VNN1 | XM_001503387 | NULL | -0.957757395 | 0.037100202 |
| ZNF211 | XM_001494387 | XP_001494437 | -0.631307968 | 0.007563067 |
| ZNF268 | XM_001494533 | XP_001494583 | -0.818874188 | 0.010320922 |
| ZNF329 | XM_001495088 | XP_001495138 | -0.652394347 | 0.043973111 |
| ZNF350 | XM_001495722 | XP_001495772 | 0.984133366 | 0.047129427 |
| ZNF784 | XM_001490354 | XP_001490404 | 1.061232126 | 0.001565597 |
| ZNRF2 | XM_001499541 | NULL | -0.636871587 | 0.018607074 |
