# Supplementary material for: Age-Related Changes following In Vitro Stimulation with Rhodococcus equi of Peripheral Blood Leukocytes from Neonatal Foals
Source: PLoS One. 2013 May 17;8(5):e62879. doi: 10.1371/journal.pone.0062879 (PMC3656898; doi:10.1371/journal.pone.0062879)
Supplement: Table S9 — List of differentially expressed genes (pvalue <0.05 and fold-change cut off of 1.5) between the stimulated leukocytes at Week-2 compared to Day 1. (DOCX) [file pone.0062879.s011.docx]

**Table S9**

| **Gene Symbol** | **NCBI accession** | **RefSeq accession** | **Log fold change** | **P-value** |
| --- | --- | --- | --- | --- |
| AADAT | XM_001498966 | XP_001499016 | -0.599841058 | 0.019685724 |
| ACADVL | XM_001504761 | XP_001504811 | -1.651823215 | 0.017418466 |
| AFF3 | XM_001492324 | XP_001492374 | -1.901706459 | 0.011746588 |
| ARFGEF1 | XM_001494559 | XP_001494609 | -1.514649073 | 0.007706563 |
| ARMC8 | XM_001496956 | XP_001497006 | -0.625057636 | 0.007458776 |
| ARPC3 | XR_036238 | NULL | -0.627643399 | 0.027328242 |
| ATP6V1C1 | XM_001494101 | XP_001494151 | -1.45038984 | 0.000571598 |
| AZIN1 | XM_001493984 | XP_001494034 | -1.004918935 | 0.047897784 |
| AZIN1 | XR_035783 | NULL | -0.795232016 | 0.004857066 |
| B3GNT2 | XM_001495317 | XP_001495367 | -1.442155249 | 0.00052999 |
| BANF2 | XM_001494025 | NULL | -0.859837701 | 0.036637416 |
| BIRC3 | XM_001499875 | XP_001499925 | -1.517216835 | 0.024332028 |
| BTBD8 | XM_001492459 | XP_001492509 | -1.415130725 | 0.009584908 |
| C12orf35 | XM_001503113 | XP_001503163 | 0.750719425 | 0.032286184 |
| C17orf56 | XM_001489994 | XP_001490044 | -0.851436055 | 0.003568557 |
| C1orf190 | XM_001495298 | XP_001495348 | -1.378103612 | 0.01276244 |
| CA12 | EF397505 | NP_001093230 | -0.608655561 | 0.005510256 |
| CBS | XM_001490849 | NULL | -1.322477266 | 0.000607311 |
| CCL20 | XM_001496798 | NULL | -2.009916209 | 0.00065999 |
| CLEC4E | XM_001492794 | XP_001492844 | -0.601616897 | 0.031106152 |
| CLINT1 | CX604855 | NULL | -0.855821145 | 0.001653176 |
| CPT1B | XM_001490719 | XP_001490769 | -1.120076377 | 0.002317933 |
| CRTC3 | XM_001498594 | XP_001498644 | 1.038409832 | 0.003098911 |
| CXCR4 | XM_001490165 | XP_001490215 | 0.979364609 | 0.00165446 |
| CYP2E1 | NM_001111303 | NULL | -0.63583653 | 0.019451826 |
| DBN1 | XM_001498360 | XP_001498410 | -1.006602239 | 0.04053832 |
| EEF1A1 | XM_001489416 | XP_001489466 | 1.119913132 | 0.001026975 |
| FAM14A | XM_001495656 | XP_001495706 | -0.644261163 | 0.008598707 |
| FAM21C | CX603317 | NULL | -1.455950406 | 0.012888314 |
| FPRL1 | XM_001497411 | XP_001497461 | -0.692646035 | 0.031678634 |
| FYB | XM_001496939 | XP_001496989 | -1.163563477 | 0.000975345 |
| GIMAP7 | NULL | NULL | 0.826658955 | 0.018917044 |
| GIMAP7 | NULL | NULL | 0.814249777 | 0.01133128 |
| GNPDA1 | XM_001503958 | XP_001504008 | -1.5737722 | 0.037194312 |
| HIP1R | XM_001492795 | XP_001492845 | -0.611008399 | 0.001101656 |
| HLA-DQB1 | XM_001492617 | XP_001492667 | 0.906897149 | 0.007864025 |
| HLA-DRA | XM_001494553 | XP_001494603 | 0.827114849 | 0.003035121 |
| IFITM1 | CD465069 | XP_001488655 | -1.310679673 | 0.011558093 |
| INDO | XM_001490681 | XP_001490731 | 1.049008421 | 0.02751077 |
| KCNK2 | XM_001488153 | XP_001488203 | -1.048962482 | 0.016000341 |
| KIF2A | XM_001493976 | XP_001494026 | -0.864738995 | 0.015725822 |
| KRT15 | XM_001496858 | XP_001496908 | -2.008355664 | 0.011479337 |

**Table S9** Continued

| **Gene Symbol** | **NCBI accession** | **RefSeq accession** | **Log fold change** | **P-value** |
| --- | --- | --- | --- | --- |
| LMOD3 | XM_001498577 | XP_001498627 | 1.96990071 | 0.007936875 |
| LOC651894 | CD466713 | NULL | -0.832969568 | 0.028204696 |
| LOC653214 | XM_001496996 | NULL | 0.598815897 | 0.00648256 |
| LOC730422 | DN507079 | NP_001108413 | -0.885191791 | 0.024265709 |
| LOC92270 | XM_001503822 | XP_001503872 | 0.628738747 | 0.001985344 |
| MPP4 | XM_001496932 | XP_001496982 | -1.312866861 | 0.005152038 |
| MRPL21 | CX604559 | XP_001499094 | -0.680057552 | 0.01325242 |
| MTHFD2L | XM_001490173 | XP_001490223 | -0.649925322 | 0.033817178 |
| NULL | CD470175 | NULL | -1.357598817 | 0.023588974 |
| NULL | CX604543 | NULL | -1.041540848 | 0.003411092 |
| NULL | CX593205 | NULL | -0.794532952 | 0.043331451 |
| OLFM4 | XM_001493449 | XP_001493499 | -0.823812846 | 0.02530932 |
| OR10G2 | XM_001498059 | XP_001498109 | -0.95779856 | 0.010799608 |
| OR2W3 | XM_001498405 | XP_001498455 | -0.781737412 | 0.003195667 |
| OR52D1 | XM_001498223 | NULL | -1.109021342 | 0.002134949 |
| PGM1 | XM_001499673 | XP_001499723 | -0.612126571 | 0.005268821 |
| PIK3AP1 | XM_001500468 | XP_001500518 | -0.758539591 | 0.001008768 |
| PLA2G5 | XM_001504348 | XP_001504398 | -1.858163792 | 0.003357951 |
| RASGEF1B | NULL | NULL | -0.679248186 | 0.01957803 |
| RETN | XM_001497441 | XP_001497491 | -0.827955159 | 0.002938616 |
| RNF144B | XM_001494349 | XP_001494399 | -0.711980364 | 0.045847975 |
| RPS25 | XM_001503063 | XP_001503113 | 0.890919203 | 0.044256309 |
| RPSA | DN504853 | NULL | 0.749437571 | 0.03611253 |
| RPSA | NULL | NULL | 0.746273817 | 0.001529256 |
| S100A12 | CD535886 | XP_001494448 | -1.532241077 | 0.026458394 |
| S100A8 | XM_001493589 | XP_001493639 | -1.417147546 | 0.01751323 |
| S100A8 | XM_001494358 | XP_001494408 | -0.982696069 | 0.047182232 |
| SDCBP | XR_036510 | NULL | -1.789133353 | 0.001510222 |
| SDCBP | XM_001496872 | XP_001496922 | -1.497244159 | 0.03342175 |
| SERPINB1 | M91161 | NP_001075416 | -1.00147438 | 0.046329777 |
| SYT2 | XM_001495156 | NULL | -1.080907123 | 0.001177288 |
| TANK | XM_001493298 | XP_001493348 | -0.818562526 | 0.030943308 |
| TFCP2 | XM_001504307 | NULL | -1.085022963 | 0.044891734 |
| TFEC | XM_001501723 | XP_001501773 | -0.798728598 | 0.000734221 |
| THBS1 | XM_001503599 | XP_001503649 | 0.624917399 | 0.024624249 |
| TMSB4X | XM_001488854 | XP_001488904 | 1.023105536 | 0.04769547 |
| TRAK1 | XM_001497614 | XP_001497664 | -0.964215761 | 0.013584595 |
| TSPO | XM_001503143 | XP_001503193 | -0.822048322 | 0.012614901 |
| TTBK2 | AB292108 | NP_001075250 | 1.096359411 | 0.047205166 |
| TTBK2 | AY237113 | NULL | 0.873142625 | 0.047749764 |

**Table S9** Continued

| **Gene Symbol** | **NCBI accession** | **RefSeq accession** | **Log fold change** | **P-value** |
| --- | --- | --- | --- | --- |
| TUBA1A | XM_001491832 | XP_001491882 | 0.580964062 | 0.004679956 |
| ZFP14 | XM_001493293 | NULL | 1.052025934 | 0.016778616 |
