# Supplementary material for: Age-Related Changes following In Vitro Stimulation with Rhodococcus equi of Peripheral Blood Leukocytes from Neonatal Foals
Source: PLoS One. 2013 May 17;8(5):e62879. doi: 10.1371/journal.pone.0062879 (PMC3656898; doi:10.1371/journal.pone.0062879)
Supplement: Table S10 — List of differentially expressed genes (pvalue <0.05 and fold-change cut off of 1.5) between the stimulated leukocytes at Week-4 compared to Day 1. (DOCX) [file pone.0062879.s012.docx]

**Table S10**

| **Gene Symbol** | **NCBI accession** | **RefSeq accession** | **Log fold change** | **P-value** |
| --- | --- | --- | --- | --- |
| ABCB10 | XM_001496372 | XP_001496422 | 1.161280858 | 0.019254903 |
| ABHD6 | XM_001489265 | XP_001489315 | 0.650778103 | 0.030679937 |
| ABI1 | XM_001494640 | XP_001494690 | -0.767737493 | 0.003962405 |
| ACADVL | XM_001504761 | XP_001504811 | -1.879594703 | 0.014211782 |
| AFF4 | XM_001504421 | XP_001504471 | -0.954288105 | 0.002818212 |
| ALPL | XM_001504312 | XP_001504362 | 0.89254865 | 0.025561207 |
| ANXA5 | XM_001503130 | XP_001503180 | -0.643605893 | 0.006481538 |
| ANXA7 | XM_001503861 | XP_001503911 | -0.650895201 | 0.035981336 |
| ARFGEF1 | XM_001494559 | XP_001494609 | -1.799871891 | 0.003416439 |
| ARHGAP1 | XM_001489971 | XP_001490021 | 1.463670456 | 0.002538143 |
| ARHGEF3 | XM_001490400 | XP_001490450 | 1.092489183 | 0.023071976 |
| ATP11B | XM_001496792 | XP_001496842 | -0.753303804 | 0.004651174 |
| ATP13A2 | XM_001488576 | XP_001488626 | 1.524868826 | 0.008006548 |
| ATP1B3 | XM_001494313 | XP_001494363 | -0.747397509 | 0.002454295 |
| ATP6V0E1 | CX602201 | XP_001502972 | 0.702958792 | 0.031368646 |
| ATP6V1C1 | XM_001494101 | XP_001494151 | -1.89525501 | 0.00024631 |
| ATXN2 | XM_001491014 | XP_001491064 | 0.964185712 | 0.010402414 |
| AZIN1 | XM_001493984 | XP_001494034 | -1.482429489 | 0.012098411 |
| AZIN1 | XR_035783 | NULL | -0.947558216 | 0.005818344 |
| BIRC3 | XM_001499875 | XP_001499925 | -1.05168096 | 0.025969003 |
| BLCAP | XM_001502351 | XP_001502401 | -0.665611044 | 0.003561071 |
| BTBD8 | XM_001492459 | XP_001492509 | -1.759125712 | 0.004509461 |
| C10orf112 | XM_001497827 | XP_001497877 | -1.069586233 | 0.006710267 |
| C14orf104 | XM_001496267 | XP_001496317 | 1.78482992 | 0.005691124 |
| C16orf78 | XM_001488683 | XP_001488733 | 1.216013897 | 0.028518142 |
| C21orf66 | XM_001494782 | XP_001494832 | 0.659321985 | 0.008569989 |
| CA12 | EF397505 | NP_001093230 | -0.829216321 | 0.036543231 |
| CACHD1 | XM_001500703 | XP_001500753 | -1.123583473 | 0.048271085 |
| CACNG1 | XM_001499847 | XP_001499897 | 0.741109289 | 0.005422058 |
| CBLB | XM_001503354 | XP_001503404 | 1.513523424 | 0.016415187 |
| CCL20 | XM_001496798 | NULL | -1.31502122 | 0.001798704 |
| CD47 | XM_001501702 | XP_001501752 | -0.62802743 | 0.020389544 |
| CDC2 | XM_001502198 | XP_001502248 | -0.757883093 | 0.002952177 |
| CDK3 | XM_001491903 | XP_001491953 | -0.84103389 | 0.011362206 |
| CHIC1 | XM_001504969 | XP_001505019 | -0.586615158 | 0.022967356 |
| CLCA2 | XM_001496218 | XP_001496268 | 1.467135987 | 0.01348303 |
| CLIC5 | XM_001502577 | XP_001502627 | 0.794455478 | 0.029767639 |
| CLINT1 | CX604855 | NULL | -0.910798085 | 0.006945032 |
| CNP | XM_001495565 | XP_001495615 | -0.662740956 | 0.011407858 |
| COL3A1 | AF117954 | NULL | 0.753124137 | 0.021760872 |
| CXXC5 | NULL | NULL | 1.237148399 | 0.023742178 |

**Table S10** Continued

| **Gene Symbol** | **NCBI accession** | **RefSeq accession** | **Log fold change** | **P-value** |
| --- | --- | --- | --- | --- |
| DPM3 | XM_001494606 | XP_001494656 | 1.407031607 | 0.020593666 |
| DZIP1 | XM_001492095 | XP_001492145 | -0.784317436 | 0.010056089 |
| EDN2 | AB079136 | NP_001075292 | -0.777616848 | 0.035669238 |
| EMR3 | XM_001495205 | XP_001495255 | -0.62598915 | 0.036379305 |
| ERCC3 | XM_001488507 | XP_001488557 | 1.171594418 | 0.032766119 |
| EXOSC4 | XR_036511 | NULL | 0.664744484 | 0.00208116 |
| FAM21C | CX603317 | NULL | -1.63507073 | 0.002486198 |
| FBXO15 | XM_001493325 | XP_001493375 | 0.761524442 | 0.02191545 |
| FPRL1 | XM_001497411 | XP_001497461 | -1.229928848 | 0.003793493 |
| FYB | XM_001496939 | XP_001496989 | -1.056103323 | 0.019260887 |
| GALNT2 | XM_001496209 | XP_001496259 | 1.678343053 | 0.021105045 |
| GK | XM_001488392 | XP_001488442 | -0.636607299 | 0.014233248 |
| GPR137 | XM_001489414 | XP_001489464 | 0.6577204 | 0.021086949 |
| GPR84 | XM_001504570 | XP_001504620 | -0.943360847 | 0.026265726 |
| HSPC152 | CX600330 | XP_001489771 | 1.125263352 | 0.048425048 |
| IFITM1 | CD465069 | XP_001488655 | -1.277246242 | 0.009153732 |
| IFNGR2 | XR_036253 | NULL | -0.654109666 | 0.019799342 |
| JAK2 | XM_001500499 | XP_001500549 | 0.629148847 | 0.034449 |
| KCNJ2 | XM_001498612 | XP_001498662 | -1.260930457 | 0.042137556 |
| KCNK2 | XM_001488153 | XP_001488203 | -0.955331639 | 0.020296789 |
| KCNK7 | XM_001493848 | XP_001493898 | 0.968250664 | 0.01938093 |
| KIAA0460 | XM_001489478 | XP_001489528 | -0.662138964 | 0.029710588 |
| KIF2A | XM_001493976 | XP_001494026 | -0.964276977 | 0.005081501 |
| KY | XM_001498469 | XP_001498519 | 0.670285539 | 0.042327107 |
| LHFPL1 | XM_001488695 | XP_001488745 | 0.722807015 | 0.010884967 |
| LOC643596 | XM_001492183 | NULL | 0.9263886 | 0.007054456 |
| LOC651894 | CD466713 | NULL | -0.952000303 | 0.000688254 |
| LOC730422 | DN507079 | NP_001108413 | -1.902043561 | 0.003593265 |
| LRMP | XM_001498588 | XP_001498638 | -0.834436784 | 0.029000685 |
| LRRC23 | XM_001497546 | XP_001497596 | 1.894459 | 0.019468393 |
| MAL | XM_001494547 | XP_001494597 | 0.917596672 | 0.005975176 |
| MAPK10 | XM_001495324 | XP_001495374 | 0.852832015 | 0.002361373 |
| MTHFD2L | XM_001490173 | XP_001490223 | -0.596670218 | 0.032990439 |
| NFKBIA | NULL | NULL | -0.770999382 | 0.019251318 |
| NICN1 | XM_001497632 | XP_001497682 | 0.728131151 | 0.002068504 |
| NT5C2 | XM_001499520 | XP_001499570 | -0.995060761 | 0.021949558 |
| NUBP1 | XR_035869 | NULL | 1.399692908 | 0.023958098 |
| NULL | CX604543 | NULL | -1.483857197 | 0.001851788 |
| NULL | DN508878 | NULL | -1.247637922 | 0.009305107 |
| NULL | CX606039 | NULL | -1.023196843 | 0.003736644 |
| NULL | XR_036509 | NULL | -0.981207915 | 0.039788418 |

**Table S10** Continued

| **Gene Symbol** | **NCBI accession** | **RefSeq accession** | **Log fold change** | **P-value** |
| --- | --- | --- | --- | --- |
| NULL | CD465425 | NULL | -0.950307718 | 0.006110222 |
| NULL | DN508978 | NULL | -0.925169145 | 0.048204521 |
| NULL | DN510428 | NULL | -0.908166329 | 0.03181999 |
| NULL | XR_036280 | NULL | -0.903209125 | 0.023578169 |
| NULL | CD469043 | NULL | -0.843652508 | 0.038041206 |
| NULL | CX602378 | NULL | -0.810701847 | 0.045863924 |
| NULL | CD528850 | NULL | -0.743187436 | 0.014108027 |
| NULL | CD528482 | NULL | -0.727772627 | 0.035418894 |
| NULL | CX601512 | NULL | -0.68960818 | 0.001654285 |
| NULL | CD468881 | NULL | -0.652960578 | 0.00062913 |
| NULL | DN507234 | NULL | -0.640263416 | 0.00768336 |
| NULL | DN508304 | NULL | -0.600152376 | 0.026311831 |
| NULL | CX602785 | NULL | -0.592140719 | 0.001939824 |
| NULL | CD468898 | NULL | -0.581370148 | 0.018228267 |
| NULL | CD470175 | NULL | 2.154640616 | 0.007565129 |
| NULL | CX605267 | NULL | 1.99496677 | 0.014646028 |
| NULL | XM_001499351 | NULL | 1.826194049 | 0.011733461 |
| NULL | XM_001497251 | NULL | 1.605571026 | 0.008928497 |
| NULL | CX600194 | NULL | 1.557605331 | 0.014299339 |
| NULL | XM_001498195 | XP_001498245 | 1.492323065 | 0.045499211 |
| NULL | DN509725 | NULL | 1.126940549 | 0.008703142 |
| NULL | XM_001502325 | XP_001502375 | 0.853830526 | 0.012400068 |
| NULL | CX603299 | NULL | 0.768572762 | 0.011388289 |
| NULL | XM_001497110 | XP_001497160 | 0.679230586 | 0.036510202 |
| NULL | CX593935 | NULL | 0.582608209 | 0.026898811 |
| OR1J4 | XM_001501393 | XP_001501443 | 0.68007645 | 0.001907715 |
| OR2S2 | XM_001493425 | XP_001493475 | 1.031860512 | 0.03330239 |
| OR52B4 | XM_001496909 | XP_001496959 | 0.740666567 | 0.006783273 |
| OR52D1 | XM_001498223 | NULL | -1.216183234 | 0.031932653 |
| OR5B2 | XM_001497992 | XP_001498042 | -0.823284405 | 0.002921001 |
| OR6C68 | XM_001504060 | XP_001504110 | -0.606378761 | 0.045664355 |
| ORM2 | XM_001488149 | XP_001488199 | -0.590570964 | 0.01759447 |
| PERQ1 | XM_001505064 | XP_001505114 | 1.305957671 | 0.001043103 |
| PGM1 | XM_001499673 | XP_001499723 | -0.850064767 | 0.004344601 |
| PICALM | XM_001490412 | XP_001490462 | -1.068610719 | 6.40E-05 |
| PIK3AP1 | XM_001500468 | XP_001500518 | -0.980084989 | 0.007484601 |
| PIM1 | XM_001500225 | XP_001500275 | 1.3762541 | 0.045883745 |
| PLA2G10 | XM_001489049 | XP_001489099 | -0.660352777 | 0.035772003 |
| PLA2G5 | XM_001504348 | XP_001504398 | -1.843177878 | 0.004780698 |
| PPP4R1 | XR_035855 | NULL | 1.147376507 | 0.000620105 |
| PRPF39 | XM_001493416 | NULL | -0.710937356 | 0.006095941 |

**Table S10** Continued

| **Gene Symbol** | **NCBI accession** | **RefSeq accession** | **Log fold change** | **P-value** |
| --- | --- | --- | --- | --- |
| RAB18 | XM_001494294 | XP_001494344 | -0.622147197 | 0.005824465 |
| RAP1B | XM_001493406 | XP_001493456 | -0.99904749 | 0.01038233 |
| RASGEF1B | NULL | NULL | -0.93326973 | 0.008852046 |
| RETN | XM_001497441 | XP_001497491 | -0.995230551 | 0.014098611 |
| RNF144A | XM_001503614 | XP_001503664 | 1.671011302 | 0.015927157 |
| RNF181 | DN504657 | NULL | 0.846822667 | 0.012766161 |
| RNF19A | XM_001492262 | XP_001492312 | -0.686283743 | 0.004148708 |
| RPAP2 | XM_001492375 | XP_001492425 | 1.387119963 | 0.024836054 |
| RTKN | XM_001500407 | XP_001500457 | 1.382375189 | 0.008040411 |
| S100P | BM734933 | XP_001501447 | -1.354950417 | 0.029387967 |
| SAA1 | NM_001081853 | NULL | -0.620685226 | 0.022778935 |
| SCG5 | XM_001501620 | XP_001501670 | -1.071491568 | 0.008619687 |
| SDCBP | XR_036510 | NULL | -1.907925119 | 0.014369212 |
| SDCBP | XM_001496872 | XP_001496922 | -1.787542663 | 0.020917757 |
| SELO | XM_001496075 | XP_001496125 | 1.211623034 | 0.009311136 |
| SERPINB1 | M91161 | NP_001075416 | -1.335315655 | 0.017392239 |
| SH3BGRL | XM_001501152 | XP_001501202 | -0.890131925 | 0.003219365 |
| SLC35D2 | XM_001494155 | XP_001494205 | 1.211854003 | 0.003445215 |
| SMARCA4 | XM_001490624 | XP_001490674 | -0.702730217 | 0.041540223 |
| SMC2 | XM_001503998 | XP_001504048 | 0.876809335 | 0.007564097 |
| SNX3 | XM_001503983 | XP_001504033 | -0.903569952 | 0.001465001 |
| SPAG4L | XM_001498564 | XP_001498614 | 0.665504638 | 0.049353951 |
| SPINLW1 | XM_001500671 | NULL | -0.605375297 | 0.026425219 |
| SUMF1 | XM_001496576 | XP_001496626 | 1.554337784 | 0.022789518 |
| TAF4B | XM_001495354 | XP_001495404 | 0.664136735 | 0.046405627 |
| TANK | XM_001493298 | XP_001493348 | -0.969842651 | 0.045667353 |
| TCTN3 | XM_001500642 | XP_001500692 | -0.876988261 | 0.004960816 |
| TFCP2 | XM_001504307 | NULL | -0.99590272 | 0.000813443 |
| TFEC | XM_001501723 | XP_001501773 | -0.895921872 | 0.003425299 |
| TLK1 | XM_001498377 | XP_001498427 | 0.930789411 | 0.005914872 |
| TMEM139 | XM_001489983 | XP_001490033 | 0.854682638 | 0.000356726 |
| TMLHE | XM_001498280 | XP_001498330 | 0.791435845 | 0.017887668 |
| TRAF3 | XM_001490000 | XP_001490050 | -0.60527754 | 0.004209033 |
| TREM1 | XM_001500981 | XP_001501031 | 0.734073069 | 0.001192172 |
| TSPO | XM_001503143 | XP_001503193 | -0.968851092 | 0.006787871 |
| UBL7 | XM_001494059 | XP_001494109 | -1.135296214 | 0.009790008 |
| UNC119 | CX601468 | XP_001504190 | 0.685298012 | 0.028585415 |
| UNC45B | NULL | NULL | 1.587404768 | 0.015616724 |
| USP22 | XM_001488603 | XP_001488653 | 0.823866683 | 0.021871856 |
| VPS54 | XM_001493998 | XP_001494048 | 1.61469506 | 0.021503377 |
| WBP1 | CX600458 | NULL | -0.79674945 | 0.012115691 |

**Table S10** Continued

| **Gene Symbol** | **NCBI accession** | **RefSeq accession** | **Log fold change** | **P-value** |
| --- | --- | --- | --- | --- |
| YIPF4 | XM_001500250 | XP_001500300 | -0.785103711 | 0.003076969 |
| ZCCHC4 | XM_001499564 | XP_001499614 | 0.630560054 | 0.01936207 |
| ZNF423 | XM_001491336 | XP_001491386 | 0.748528672 | 0.02733138 |
| ZNF462 | XM_001493275 | XP_001493325 | 1.446451666 | 0.015667213 |
