# Supplementary material for: Age-Related Changes following In Vitro Stimulation with Rhodococcus equi of Peripheral Blood Leukocytes from Neonatal Foals
Source: PLoS One. 2013 May 17;8(5):e62879. doi: 10.1371/journal.pone.0062879 (PMC3656898; doi:10.1371/journal.pone.0062879)
Supplement: Table S11 — List of differentially expressed genes (pvalue <0.05 and fold-change cut off of 1.5) between the stimulated leukocytes at Week-8 compared to Day 1. (DOCX) [file pone.0062879.s013.docx]

Table S11

| **Gene Symbol** | **NCBI accession** | **RefSeq accession** | **Log fold change** | **P-value** |
| --- | --- | --- | --- | --- |
| ACADVL | XM_001504761 | XP_001504811 | -2.087284003 | 0.000847443 |
| ARFGEF1 | XM_001494559 | XP_001494609 | -1.610113438 | 0.003378656 |
| ARRB2 | XM_001502964 | XP_001503014 | 0.606058556 | 0.043532194 |
| ASNSD1 | XM_001499046 | XP_001499096 | 1.077796478 | 0.033549869 |
| ATP11B | XM_001496792 | XP_001496842 | -0.822230159 | 0.016452645 |
| ATP1B3 | XM_001494313 | XP_001494363 | -0.648695613 | 0.005482556 |
| ATP6V1C1 | XM_001494101 | XP_001494151 | -1.202587628 | 0.034009971 |
| ATRX | XM_001502685 | XP_001502735 | -0.616259946 | 0.039278326 |
| BCL2A1 | XM_001487956 | XP_001488006 | 0.955041415 | 0.044290947 |
| BTBD8 | XM_001492459 | XP_001492509 | -0.924766866 | 0.038558138 |
| C11orf58 | XR_036382 | NULL | 0.653065607 | 0.023198729 |
| C7orf34 | XM_001490979 | NULL | 0.601862595 | 0.046129133 |
| CASP4 | XM_001499070 | XP_001499120 | 0.600992949 | 0.046434391 |
| CCL20 | XM_001496798 | NULL | -0.778462967 | 0.005110509 |
| CD69 | XM_001499388 | XP_001499438 | 0.740935237 | 0.004646201 |
| CDK3 | XM_001491903 | XP_001491953 | -0.664564519 | 0.04827149 |
| CLINT1 | CX604855 | NULL | -0.981385555 | 0.006492774 |
| DEK | XM_001496593 | XP_001496643 | 0.931423833 | 0.015238457 |
| DNAJC10 | XM_001498066 | XP_001498116 | 0.77186899 | 0.041010184 |
| EEF1A1 | NULL | NULL | 1.095967951 | 0.024315896 |
| EHHADH | XM_001498736 | XP_001498786 | -0.766644773 | 0.026279904 |
| EML3 | XM_001494714 | XP_001494764 | 0.791210587 | 0.019950462 |
| EMR3 | XM_001495205 | XP_001495255 | -0.646968248 | 0.030763809 |
| FAM21C | CX603317 | NULL | -1.555535197 | 0.048848611 |
| FAM3A | XM_001492203 | XP_001492253 | -0.945540312 | 0.040937953 |
| FAM45A | CX598831 | NULL | 0.639509616 | 0.033204238 |
| FGF17 | XM_001490190 | XP_001490240 | 0.592744908 | 0.029997885 |
| FOXN1 | XM_001504134 | XP_001504184 | -0.689854717 | 0.027621125 |
| FYB | XM_001496939 | XP_001496989 | -0.982925809 | 0.020545064 |
| GIMAP7 | NULL | NULL | 1.04459567 | 0.010520798 |
| GIMAP7 | NULL | NULL | 0.825287594 | 0.0283829 |
| GPR84 | XM_001504570 | XP_001504620 | -0.620613813 | 0.031937423 |
| GRHL2 | XM_001493287 | XP_001493337 | -0.821895236 | 0.006017268 |
| HLA-DQB1 | XM_001492617 | XP_001492667 | 0.800585287 | 0.000840462 |
| HLA-DRA | XM_001494553 | XP_001494603 | 0.893882438 | 0.023371877 |
| HLA-DRB1 | XM_001495531 | XP_001495581 | 0.68056067 | 0.027745738 |
| HOXA11 | XM_001499702 | NULL | 0.683219088 | 0.039182344 |
| HSPA8 | AF411802 | NULL | 1.058149497 | 0.033203756 |
| IFITM1 | CD465069 | XP_001488655 | -1.11443803 | 0.022143739 |
| IL7R | EF035169 | NP_001075411 | 0.685082157 | 0.03278201 |
| INDO | XM_001490681 | XP_001490731 | 1.481234967 | 0.019946121 |

**Table S11** Continued

| **Gene Symbol** | **NCBI accession** | **RefSeq accession** | **Log fold change** | **P-value** |
| --- | --- | --- | --- | --- |
| KIF2A | XM_001493976 | XP_001494026 | -1.306312758 | 0.008457702 |
| LOC339457 | XM_001495309 | NULL | -0.653234193 | 0.022665678 |
| LOC651894 | CD466713 | NULL | -0.930651515 | 0.02515162 |
| LOC653214 | XM_001496996 | NULL | 0.63790332 | 0.016630539 |
| LOC730422 | DN507079 | NP_001108413 | -1.396569217 | 0.005032256 |
| MAP4K4 | XM_001491720 | XP_001491770 | 0.786029617 | 0.027023591 |
| MC3R | XM_001489123 | XP_001489173 | 0.794228461 | 0.035617852 |
| MMP1 | AF148882 | NP_001075316 | -1.383265349 | 0.018457835 |
| NULL | CD528482 | NULL | -1.001419634 | 0.036841621 |
| NULL | CX606039 | NULL | -0.950759774 | 0.01062028 |
| NULL | CD466166 | NULL | -0.825130352 | 0.036207037 |
| NULL | CX604543 | NULL | -0.749051439 | 0.036238202 |
| NULL | CX602785 | NULL | -0.61274206 | 0.0012007 |
| NULL | CD469036 | NULL | -0.612438036 | 0.024935751 |
| NULL | CX593205 | NULL | -0.610547975 | 0.02273951 |
| NULL | CD528850 | NULL | -0.604770805 | 0.046956995 |
| NULL | DN508293 | XP_001504999 | 1.64644475 | 0.02100169 |
| NULL | CD465991 | XP_001503681 | 1.258970823 | 0.006486919 |
| NULL | CD465610 | NULL | 0.972664732 | 0.022041409 |
| NULL | CD536657 | NULL | 0.938773528 | 0.039296938 |
| NULL | CX605248 | NULL | 0.905460231 | 0.03371701 |
| NULL | DN507426 | NULL | 0.772989861 | 0.048495289 |
| NULL | CD469128 | NULL | 0.608263541 | 0.000200687 |
| NULL | DN509978 | NULL | 0.597142053 | 0.040207122 |
| OR52D1 | XM_001498223 | NULL | -0.6527913 | 0.018588612 |
| OR5B2 | XM_001497992 | XP_001498042 | -1.06714031 | 0.044328516 |
| ORM2 | CD536428 | XP_001488234 | 0.743300485 | 0.049920887 |
| OXTR | XM_001491665 | XP_001491715 | -0.680126049 | 0.047532481 |
| PAIP1 | NULL | NULL | 0.665814841 | 0.01745315 |
| PGM1 | XM_001499673 | XP_001499723 | -0.737188302 | 0.00474333 |
| PLA2G5 | XM_001504348 | XP_001504398 | -1.43726865 | 0.015664865 |
| PRPF39 | XM_001493416 | NULL | -0.846397133 | 0.003751746 |
| PTPRZ1 | XM_001501199 | XP_001501249 | -0.674121723 | 0.019505069 |
| RAD50 | XM_001504442 | XP_001504492 | -0.658976212 | 0.021970309 |
| RASSF2 | BM735017 | NULL | -0.586614888 | 0.026433264 |
| RHOG | XM_001496655 | XP_001496705 | -0.877560931 | 0.034669375 |
| RNF149 | XM_001491820 | XP_001491870 | -0.633153847 | 0.048241295 |
| RNF17 | XM_001488530 | XP_001488580 | -0.613166043 | 0.032459796 |
| RPL10 | NULL | NULL | 0.860666446 | 0.025198181 |
| RPL28 | CD464415 | XP_001495815 | 0.855569252 | 0.023662502 |
| RPL31 | XM_001489469 | XP_001489519 | 1.146218833 | 0.010572086 |
| RPL36A | NULL | NULL | 0.701082298 | 0.003860024 |

**Table S11** Continued

| **Gene Symbol** | **NCBI accession** | **RefSeq accession** | **Log fold change** | **P-value** |
| --- | --- | --- | --- | --- |
| RPL37A | XM_001500265 | XP_001500315 | 0.784064991 | 0.014120044 |
| RPL7 | XM_001491982 | XP_001492032 | 1.389483366 | 0.030492423 |
| RPLP1 | BM735439 | XP_001495775 | 0.791525336 | 0.016447705 |
| RPS2 | XR_036216 | NULL | 0.626066785 | 0.01084341 |
| RPS25 | XM_001503063 | XP_001503113 | 1.365923741 | 0.028074188 |
| RPS3A | XM_001501474 | XP_001501524 | 1.350921423 | 0.044803898 |
| RPSA | XM_001497103 | XP_001497153 | 1.331451612 | 0.041836193 |
| S100A13 | CX601420 | XP_001494839 | 0.66414564 | 0.037215882 |
| S100A8 | XM_001493589 | XP_001493639 | -1.413323341 | 0.015480451 |
| S100A8 | XM_001494358 | XP_001494408 | -0.801027911 | 0.016183703 |
| SCYL1 | CX602064 | NULL | -0.790557375 | 0.046564998 |
| SDCBP | XR_036510 | NULL | -1.077260645 | 0.0490504 |
| SEC61G | XM_001498680 | NULL | 0.759066217 | 0.036271045 |
| SERPINB1 | M91161 | NP_001075416 | -1.145077426 | 0.030318397 |
| SGCE | XM_001493182 | XP_001493232 | -0.644246152 | 0.030496341 |
| SHFM1 | XM_001494095 | XP_001494145 | 0.616331664 | 0.014870371 |
| SNX3 | XM_001503983 | XP_001504033 | -0.732013342 | 0.041336429 |
| SPCS1 | XM_001501306 | NULL | 0.589014524 | 0.032431208 |
| TFCP2 | XM_001504307 | NULL | -0.687762496 | 0.034288891 |
| TFEC | XM_001501723 | XP_001501773 | -0.604147246 | 0.016938484 |
| TOR2A | XM_001501467 | XP_001501517 | 1.14341584 | 0.016151657 |
| TOR3A | XM_001494447 | XP_001494497 | -0.922058318 | 0.026579156 |
| TRIM47 | DN510014 | NULL | -0.688295223 | 0.019260471 |
| TSPO | XM_001503143 | XP_001503193 | -0.706198165 | 0.04877139 |
| TUBA1A | XM_001491832 | XP_001491882 | 0.865009711 | 0.014219178 |
| UBL7 | XM_001494059 | XP_001494109 | -1.243838638 | 0.016938105 |
| USP48 | XM_001504296 | XP_001504346 | -0.730407902 | 0.002124971 |
| WIPI1 | XM_001499325 | XP_001499375 | -0.689463493 | 0.025966418 |
